# Base editing rescues seizures and sudden death in a *SCN8A* mutation-associated developmental epileptic encephalopathy model

**DOI:** 10.1172/JCI196402

**Published:** 2026-02-02

**Authors:** Caeley M. Reever, Alexis R. Boscia, Tyler C.J. Deutsch, Mansi P. Patel, Raquel M. Miralles, Shrinidhi Kittur, Erik J. Fleischel, Atum M.L. Buo, Matthew S. Yorek, Miriam H. Meisler, Charles R. Farber, Manoj K. Patel

**Affiliations:** 1 Department of Anesthesiology, University of Virginia Health System, Charlottesville, Virginia, USA.; 2 Neuroscience Graduate Program,; 3 Department of Neuroscience, and; 4 Department of Genomic Sciences, University of Virginia; Charlottesville, Virginia, USA.; 5 Department of Human Genetics, University of Michigan, Ann Arbor, Michigan, USA.

**Keywords:** Genetics, Neuroscience, Epilepsy, Gene therapy, Sodium channels

## Abstract

*SCN8A* encodes the voltage-gated sodium channel Na_v_1.6, which plays a key role in facilitating neuronal excitability. Mutations in *SCN8A*, particularly gain-of-function variants, cause *SCN8A* developmental and epileptic encephalopathy (DEE), a severe epilepsy syndrome characterized by seizures, cognitive dysfunction, movement disorders, and sudden unexpected death in epilepsy (SUDEP). The recurrent *SCN8A* variant R1872W impairs channel inactivation, causing neuronal hyperexcitability and seizures. Current treatments, including antiseizure medications, are often ineffective for patients with *SCN8A* DEE, highlighting the need for targeted therapies. We employed base editing to correct the R1872W *SCN8A* variant. An adenine base editor and guide RNA (*SCN8A*-ABE) were packaged within dual PhP.eB-adeno-associated viruses (AAVs) and administered to R1872W mice at P2. *SCN8A*-ABE significantly increased survival of mice expressing R1872W and either reduced seizure incidence and severity or eliminated seizure occurrence. Electrophysiological recordings revealed a rescue of seizure-associated neuronal hyperexcitability and suppression of the pathogenic persistent sodium current (I_NaP_) in treated mice. Comorbidities, including diminished mobility and anxiety-like behaviors, were improved by *SCN8A-*ABE. These effects were achieved by a 32% absolute reduction in mutant transcripts, accompanied by conversion to *SCN8A* WT transcripts. Our findings demonstrate base editing as an effective targeted therapeutic approach for *SCN8A* DEEs by addressing the underlying genetic cause.

## Abstract 2

A gene therapy targeting a deadly mutation causing epilepsy rescues seizures and disease symptoms, offering a promising gene therapy for other genetic forms of epilepsy.

## Introduction

*SCN8A* encodes the voltage-gated sodium channel Na_v_1.6, which is expressed primarily in the central and peripheral nervous systems (CNS and PNS, respectively) and is essential for the initiation and propagation of action potentials in excitable cells (1). It is highly expressed in both excitatory and inhibitory neurons and found concentrated at the nodes of Ranvier and the axon initial segment (AIS) (2). Many pathogenic variants in *SCN8A* lead to developmental and epileptic encephalopathy (DEE) (3–7). To date, over 800 individuals have been identified with pathogenic *SCN8A* variants, and many exhibit a gain-of-function (GOF) phenotype (5, 6). Patients experience refractory seizures and a range of comorbidities, including movement disorders, developmental delays, and cognitive dysfunction. The risk of sudden unexpected death in epilepsy (SUDEP) is high in the *SCN8A* DEE patient population (7). A recurrent single nucleotide polymorphism (SNP) at residue 1872 accounts for approximately 10%–15% of all *SCN8A* DEE cases and is associated with neonatal seizures (5, 6, 8). The CGG codon for arginine, at residue 1872, contains a CpG dinucleotide, which is prone to a high mutation rate due to its vulnerability to cytosine deamination (9). In Na_v_1.6 channels harboring the R1872W (c.5614C>T) mutation, the loss of arginine in the C-terminal domain destabilizes its interaction with the inactivation gate, resulting in altered sodium channel inactivation and increased neuronal excitability that underlies the severe seizure phenotype in *SCN8A* DEE (8, 10–12). Increases in the persistent sodium channel current (I_NaP_) have been identified as a major pathological current driving epileptiform activity in many epileptic encephalopathy-causing variants, including the *SCN8A* R1872W variant (8, 10, 13). Current treatment options are limited to the use of anti-seizure medications (ASMs) that target sodium channels as a means of suppressing seizure activity without affecting the underlying genetic cause. Unfortunately, many patients are treatment resistant while others suffer intolerability issues and/or side effects associated with ASMs (14, 15).

Base editing is a CRISPR/Cas9–derived genome editing technology that facilitates the direct and irreversible conversion of one DNA nucleotide into either a cytosine or adenine without inducing double-stranded DNA breaks (16, 17). The foundational concept involves fusing a catalytically impaired or “dead” Cas9 to a DNA-modifying enzyme, or deaminase, which is precisely guided to a target locus by a guide RNA (sgRNA). Initial discovery of base editing came with the development of Cytosine Base Editors (CBEs), which enable C-to-T transitions via the deamination of cytosine to uridine, subsequently recognized as thymine during DNA replication or repair (16). The precise conversion of A•T base pairs to G•C base pairs was discovered through directed evolution, leading to the engineering of Adenine Base Editors (ABEs) (17). ABEs consist of an engineered *E*. *coli* tRNA adenosine deaminase (TadA) evolved to deaminate adenine within DNA. ABEs target adenine (A) converting it to inosine (I), which is then read as guanine (G) by DNA polymerases during replication or repair.

Here, we have used adenine base editors (ABEs) (17, 18) to precisely revert the SNP (c.5614C>T) at the 1872 residue of *SCN8A* from T/A to C/G, thereby addressing the underlying cause of the disorder. We identified a highly effective construct and its paired editor for correction of R1872W, referred to as *SCN8A*-ABE. In vivo, S*CN8A*-ABE treatment increased survival and either significantly reduced seizure frequency and intensity or completely abolished seizure onset in a mouse model of *SCN8A* R1872W DEE. *SCN8A*-ABE treatment resulted in A-to-G editing efficiencies observable in RNA transcripts of approximately 32% absolute reduction of mutant transcripts, reverting the mutant tryptophan (W; TGG) to WT arginine (R; CGG). No significant off-target editing (≤ 1%) was observed in any adenines in a total 290 predicted potential off-target sites in transcriptome-wide RNA-seq and Whole Genome Sequencing (WGS). Electrophysiology recordings revealed attenuation of neuronal hyperexcitability and a suppression of aberrant I_NaP_ activity in *SCN8A*-ABE treated mice. Additionally, we observed mitigation of multiple behavioral abnormalities associated with *SCN8A* DEE after *SCN8A*-ABE treatment in mice. In summary, we demonstrate that base editing therapy rescues the disease phenotype of *SCN8A* DEE by targeting the underlying genetic cause and ameliorating key aspects of the disorder. These studies highlight the immense potential of base editing techniques as a therapeutic approach for not only *SCN8A* DEE, but for other genetic epilepsies as well.

## Results

### Design and validation of adenine base editor targeting SCN8A R1872W.

**Figure 1:**
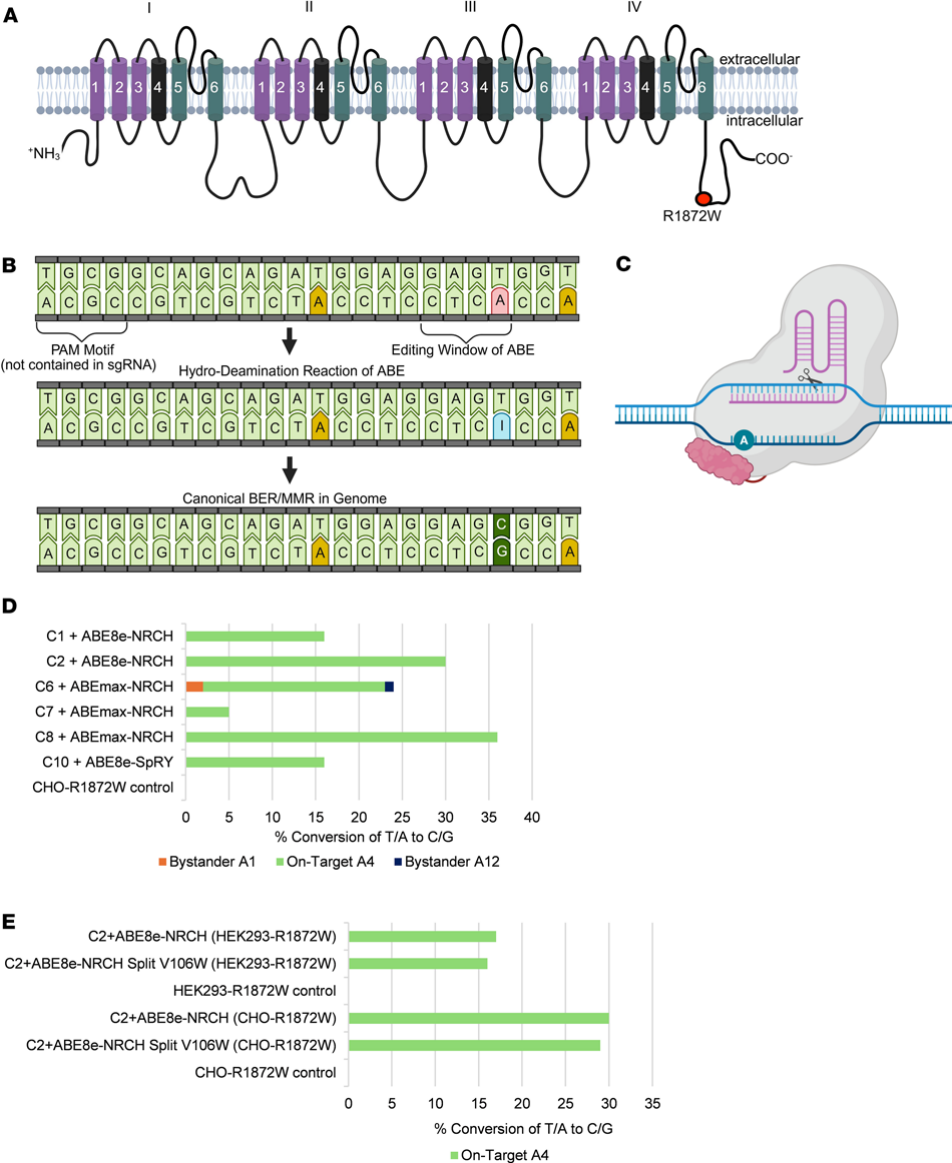
Comparative analysis of on-target ABE efficiencies in cell lines. (**A**) Location of the R1872W variant (red) in the C-terminal domain of the Na_v_1.6 voltage gated sodium channel. (**B**) Adenine base editing at the R1872W locus. Target adenine (red) at the first position of the tryptophan codon is converted to inosine (blue) by the *SCN8A*-ABE and subsequently to guanine and cytosine pairing (dark green) via base excision and mismatch repair. Potential bystander adenines in the target sequence are depicted in yellow. (**C**) Cartoon depicting an adenine base editor. (**D**) Multiple constructs were screened in CHO-R1872W rodent cells. The efficacy of various base editors for on-target adenine conversion was assessed by Sanger sequencing. A4 on-target, green; A1 bystander, orange; A12 bystander, navy. (**E**) Construct C2 was optimized for on-target adenine conversion rates and minimal bystander activity in CHO-R1872W cells and HEK293-R1872W cells with the addition of a split intein structure and a V106W mutation in the TadA-8e deaminase domain. A4, green; on-target. Outcomes were assessed by Sanger sequencing.

The *SCN8A* variant R1872W (c.5614C>T) (Figure 1A) results from a CGG to TGG substitution on the coding DNA strand. We targeted the negative (complementary) strand to enable an adenine to guanine (A-to-G) conversion. This ultimately resulted in a TGG to CGG correction upon completion of the intermediate base editing conversion of adenine to inosine (Figure 1, B and C) (17). Additionally, there is no canonical NGG PAM, the typical recognition site for Cas9 (19), near the R1872W loci in a utilizable position for single guide RNA(sgRNA) constructs. Therefore, we explored base editors with fluid PAM sequences in cell screens to identify the most efficient constructs. We designed a set of sgRNA sequences capable of utilizing 23 published ABE deaminase constructs with diverse PAM recognition profiles to revert R1872W. We chose to pair these into 16 testable constructs (Supplemental Table 1 and Supplemental Figure 1; supplemental material available online with this article; https://doi.org/10.1172/JCI196402DS1) based on reported PAM preferences (20–23) and high predictive accuracy from BE-HIVE (24), CRISPOR (25), and CRISPR RGEN tools (26). Each construct was designed to target the same adenine at the R1872W *SCN8A* locus and took into consideration the available PAM sequences in the area.

To screen for efficient base editing, sgRNA constructs were initially tested in HEK293 cells engineered to harbor a mouse codon-optimized *SCN8A* R1872W allele (HEK293-R1872W). These cells were selected for their ease of transfection and editing efficiencies, which were determined in our experiment by genomic DNA extraction and Sanger sequencing without the need for antibiotic selection or flow cytometry GFP sorting. Among the 16 tested constructs, 4 constructs achieved acceptable A-to-G conversion (≥ 15%) without A-to-G bystander editing (≤ 1%). Another 4 constructs exhibited bystander adenine editing, and 8 constructs showed no evident on-target A-to-G conversion, disqualifying guides under both categories from further in vitro studies. (Supplemental Figure 1). To mitigate the effects of HEK293 cell triploidy and account for species-specific genomic codon differences influencing guide RNA efficiency, the most effective constructs with no bystander A-to-G activity were evaluated in CHO Flp-In cells (CHO-R1872W). The CHO line provides a diploid genomic context more representative of typical mammalian somatic karyotypes in mice and humans, unlike the multiallelic integration in HEK293 cells (27, 28). These cells, engineered to carry the same *SCN8A* R1872W allele, provide a diploid genomic context and serve as a rodent model proxy for in vivo applications. Editing efficiencies in CHO-R1872W cells were similar to those observed in HEK293 cells with 4 constructs achieving acceptable A-to-G conversion (≥ 15%) with only 1 construct exhibiting potential bystander adenine editing (Figure 1D). Guide C2 and ABE8e-NRCH exhibited robust activity in the murine genome. We selected C2 for in vivo analyses due to the absence of nearby bystander adenines within the immediate target window (4–7 nucleotides (nt) from 5’ start of guide) (19). Additionally, its paired editor demonstrated enhanced fidelity compared with prior ABEs (19) along with compatibility to undergo evolution to recognize PAM sequences such as NRCH (21), where *N =* any nucleotide, R = A or G, and H = A, C, or T.

The introduction of a V106W mutation into the deaminase domain of ABE8e achieves significantly reduced DNA off-target effects (19, 29). The V106W modified deaminase domain has shown to be particularly advantageous in gene editing scenarios in which minimizing off-target and bystander activity is crucial. This modification would be advantageous for the R1872W mutation, where the presence of multiple adenines in the target sequence poses a heightened risk in a sensitive pediatric population. We introduced the V106W mutation into the base editor; the construct was then divided into N-terminal and C-terminal pAAV backbones, incorporating NpuN and NpuC intein proteins as previously described (30, 31) to form the C2 and ABE8e-NRCH V106W split intein construct (C2 Split-V106W) (Figure 1E). To assess whether these modifications affected construct activity, we screened them in the same engineered HEK293-R1872W and CHO-R1872W cell lines, comparing results to C2 paired with unmodified ABE8e-NRCH. C2 Split-V106W exhibited no significant on-target reduction at the R1872W locus editing compared with C2 paired with unmodified ABE8e-NRCH in both cell lines (Figure 1E).

### SCN8A-ABE treatment ameliorates seizure activity and premature death in R1872W-expressing mice.

**Figure 2:**
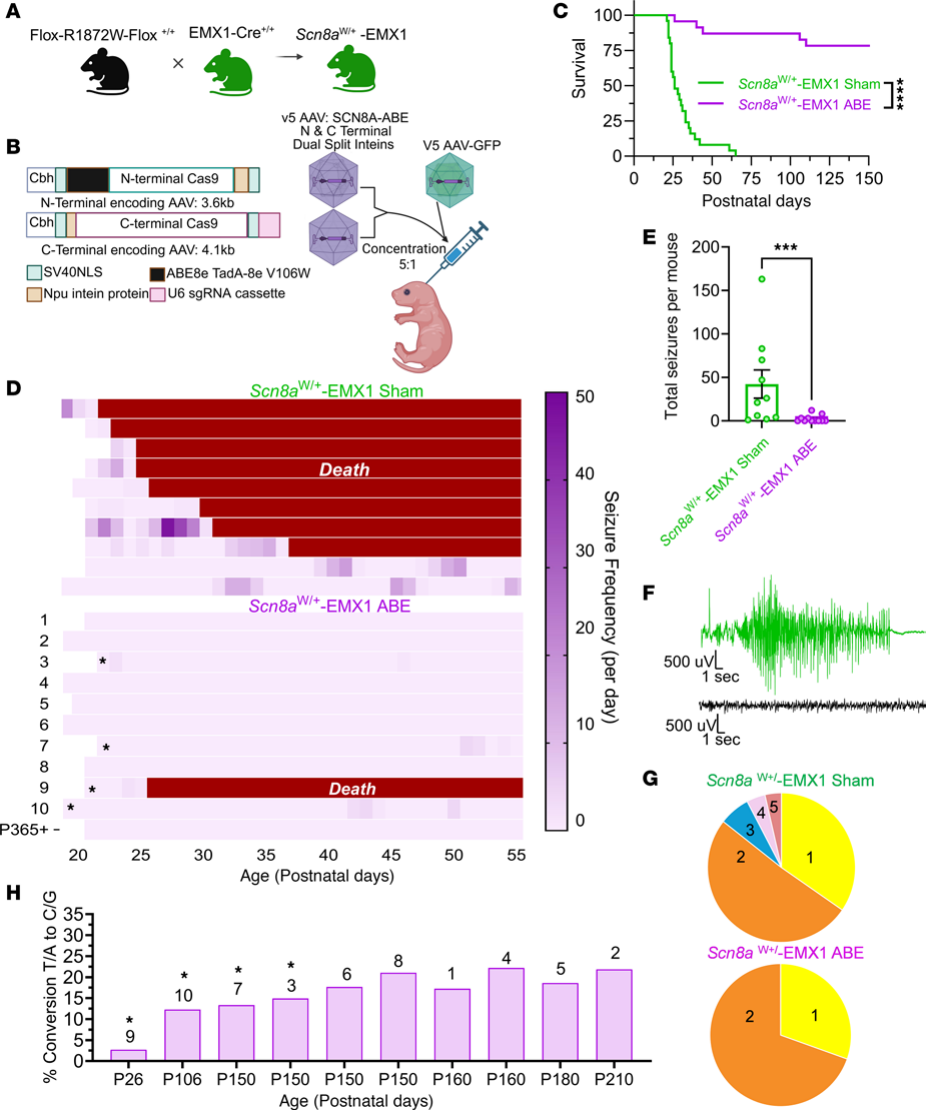
Treatment with *SCN8A*-ABE significantly increases survival and reduces seizure frequency. (**A**) Breeding strategy to generate *Scn8a*^W/+^-EMX1 mice. (**B)** P2 mice were injected intraventricularly (ICV) with a 5:1 ratio of AAV *SCN8A*-ABE to AAV-GFP with a total of 1.0 × 10^11^ viral genomes (vg) of the *SCN8A*-ABE treatment (5.0 × 10^10^ vg of each dual intein PhP.eB-ABE vectors along with 2.0 × 10^10^ vg PhP.e-GFP). Littermates were injected only with 2.0 × 10^10^ vg of PhP.e-GFP as a ‘sham’ viral transduction control. (**C**) Survival of *Scn8a*^W/+^-EMX1 mice is significantly increased with *SCN8A*-ABE treatment (purple, *n =* 23) compared with sham control *Scn8a*^W/+^-EMX1 mice (green, *n =* 25, *P* < 0.0001, Log-rank Mantel-Cox test). (**D**) Spontaneous seizure incidence (purple shading) in sham control (*n =* 10) and *SCN8A*-ABE treated (*n =* 11) *Scn8a*^W/+^-EMX1 mice over a period of 35 days. Red bar indicates seizure-induced death. In this panel (**D**), * indicates *Scn8a*^W/+^-EMX1 *SCN8A*-ABE–treated mice that experienced seizures, which correlate with panel **H**. (**E**) Sham control *Scn8a*^W/+^-EMX1 mice (*n =* 10) exhibit significantly more seizures than their *SCN8A*-ABE–treated counterparts (*n =* 11); (***P < 0.001, Mann-Whitney test). Bars represent mean ± SEM. (**F**) Representative EEG trace of a stage 5 Racine scale seizure recorded from a sham control *Scn8a*^W/+^-EMX1 mouse (green). Mouse succumbed to seizure-induced death immediately following the seizure. Bottom trace, (black) shows example trace of EEG activity from a *Scn8a*^W/+^-EMX1 *SCN8A*-ABE–treated mouse. (**G**) Severity of seizures recorded from *Scn8a*^W/+^-EMX1 *SCN8A*-ABE–treated mice (*n =* 3 mice, *n =* 23 seizures) compared with *Scn8a*^W/+^-EMX1 sham control mice (*n =* 9 mice, *n =* 416 seizures). Numbers represent Racine scale seizure classification. (**H**) Percentage of T-to-C conversion of the mutant R1872W allele in *Scn8a* ^W/+^-EMX1 *SCN8A*-ABE–treated mice (*n =* 10) assessed for seizure activity at varying time points. Numbers shown correlate T-to-C conversion with effects on seizure incidence for mice shown in **D**. Asterisks indicate mice that exhibited seizure activity in **D**.

To assess the efficacy of our base editors in vivo, we used a mouse model in which the corresponding site in mouse *SCN8A* has been mutated to create the human R1872W missense mutation in a Cre-dependent manner (*Scn8a*^W/+^) (10, 32). We used EMX1-Cre to generate a *SCN8A* DEE mouse model expressing the R1872W variant exclusively in forebrain excitatory neurons (*Scn8a*^W/+^-EMX1) (Figure 2A) (10). *Scn8a*^W/+^-EMX1 mice exhibit a typical seizure onset around P20 (10, 33), allowing time for peak expression of our base editing AAVs and correction of the variant (34, 35).

To achieve in vivo base editing of the mutant R1872W allele, we designed a dual intein adeno-associated virus (AAV) delivery strategy, referred to as *SCN8A*-ABE, to package the split ABE8e-NRCH (V106W) base editor paired with the C2 sgRNA (C2 Split-V106W) into 2 AAV capsids, as previously described (31) (Figure 2B). The *SCN8A*-ABE treatment of dual intein vectors was paired with a third AAV directing GFP expression to visualize treated cells in later experiments (31). For packaging, we selected the AAV.PhP.eB capsid, an engineered variant of AAV9 (36–38). We utilized PhP.eB to deliver the *SCN8A*-ABE treatment along with the partnered GFP virus. As a control, we used the partnered GFP virus in the absence of *SCN8A*-ABE, referred to as sham control (31).

We delivered either *SCN8A*-ABE or sham virus via ICV injection at P2 in *Scn8a*^W/+^-EMX1 mice, (Figure 2B). *SCN8A*-ABE treatment significantly prolonged the survival of approximately 87% of *SCN8A*-ABE–treated mice (*n =* 23) compared with sham control *Scn8a*^W/+^-EMX1 mice (*n =* 25) (Figure 2C). Early mortality was observed in 3 *SCN8A*-ABE–treated *Scn8a*^W/+^-EMX1 mice (< P65). An additional 2 ABE-treated *Scn8a*^W/+^-EMX1 mice survived until P106 and P110. For sequencing purposes, we euthanized 16 *SCN8A*-ABE–treated mice at varying timepoints after P150, making the true survival rates for *SCN8A*-ABE–treated *Scn8a*^W/+^-EMX1 mice impossible to accurately calculate (Figure 2C and Supplemental Figure 2, C and D).

Increased survival in *SCN8A*-ABE–treated mice was likely attributed to a decrease in seizure burden. To assess this, we evaluated seizures using concurrent video and electroencephalogram (EEG) recordings in *Scn8a*^W/+^-EMX1 mice treated either with *SCN8A*-ABE (*n =* 11) or the sham virus (*n =* 10) (Figure 2, D and E and Supplemental Video 1). Spontaneous seizures were detected in all sham control *Scn8a*^W/+^-EMX1 mice, and 8 of 10 mice succumbed to seizure-induced death before P40. The 2 remaining sham control *Scn8a*^W/+^-EMX1 mice succumbed to death before P65. We evaluated seizure severity using the standard Racine Scale. Sham control *Scn8a*^W/+^-EMX1 mice experienced 1–5 Racine scale seizures (Figure 2G). In contrast, spontaneous seizures were completely inhibited in 7 of the 11 *SCN8A*-ABE–treated *Scn8a*^W/+^-EMX1 mice (Figure 2, D and E). In another 3 mice, seizure frequency was significantly reduced, and seizure severity was reduced to only stage 1–2 Racine scale seizures (Figure 2, F and G).

Global expression of the R1872W variant via EIIa-Cre (*Scn8a*^W/+^-EIIa) leads to premature death at approximately P15 (10). We also evaluated our base editor in these mice (*n =* 12; Supplemental Figure 4). Peak expression of AAVs, and therefore editing activity, occurs around 3 weeks (34, 35, 39). Despite the severity and timeline of seizure-related death in this expression model, *SCN8A*-ABE treatment significantly increased the average survival of approximately half of all *SCN8A*-ABE–treated *Scn8a*^W/+^-EIIa mice. Two *SCN8A*-ABE–treated mice were euthanized for sequencing at P163 and P283, therefore also underestimating the true survival time (Supplemental Figure 4, B, D, and E). Sham control *Scn8a*^W/+^-EIIa mice (*n =* 13) survived to an average age of P14.5, typically following a single seizure event.

### ABE treatment demonstrates high R1872W loci–targeting efficacy.

We used Next-Generation targeted amplicon Sequencing (NGS) to assess the extent of correction of R1872W variant alleles from mice used to generate the survival curves. This was detected via PCR primers spanning the TALEN-silent mutations inserted within the mutant R1872W allele only (10, 32, 40) (Supplemental Figure 2A). For this sequencing process, we leveraged these engineered silent substitutions, introduced originally to prevent TALEN recleavage, to enabled selective amplification and quantification of exon 26b, the only locus harboring the pathogenic R1872W variant (CGG to TGG on the + strand; CCA on the – strand) (10, 32). Notably, while exon 26a and the WT allele contain adenines at positions A1 and A12 (corresponding to A2 and A13 in guide RNA alignment), they lack the critical A4 (A5 in chosen guide coordinates), which defines the targetable mutant site. Thus, A4 editing serves as a unique marker for the pathogenic exon 26b, while A1 and A12 represent potential bystander edits across all three alleles.

We dissected hippocampal and cortical regions from 21 total *SCN8A*-ABE treated *Scn8a*^W/+^-EMX1 mice and 19 sham control *Scn8a*^W/+^-EMX1 mice assessed for survival in Figure 2C. In *SCN8A*-ABE–treated *Scn8a*^W/+^-EMX1 mice that did not exhibit premature death and survived past timepoint P150 (*n =* 16), we observed a reversion of 18.5% of the mutant allele T/A to WT C/G in genomic DNA compared with sham control *Scn8a*^W/+^-EMX1 mice (from 72.5% ± 1.7% (*n =* 19) in sham control to 54.0% ± 0.9% (*n =* 16) in *SCN8A*-ABE–treated mice). The percentage of reads from all cells in these regions with the variant T/A at the R1872W locus of *SCN8A* was reduced and WT base pairing of C/G at the R1872W locus increased with an equivalent percentage (Supplemental Figure 2B). Additional sequencing revealed less than 5% editing within the hippocampus and cortex of 3 *SCN8A*-ABE–treated *Scn8a*^W/+^-EMX1 mice that experienced premature death (P26, P40, and P44; Figure 2, C and H, and Supplemental Figure 2, C and D). Thus, the observed level of editing in these mice was insufficient to support survival. On target Na_v_1.6 A4 mRNA sequencing of *Scn8a*^W/+^-EMX1 *SCN8A*-ABE–treated mice (*n =* 13) that were euthanized after P150 shows approximately 32% on-target A-to-G reversion in RNA of mutant transcript (from 43.4% ± 1.9% in sham controls (*n =* 5) to 11.6% ± 1.5% in *SCN8A*-ABE–treated mice (*n =* 13); Supplemental Figure 2E). The correlation between transcript expression and seizures is shown in Supplemental Table 2. DNA editing efficiencies above 15% were associated with complete seizure cessation and the prevention of seizure-induced death.

We also assessed the extent of correction of the R1872W mutation in dissected hippocampal and cortical regions from 11 *SCN8A*-ABE–treated *Scn8a*^W/+^-EIIa mice and 9 sham control *Scn8a*^W/+^-EIIa mice used to generate the survival curve shown in Supplemental Figure 4B. We observed approximately 20% reversion in the mutant allele of T/A to WT C/G in genomic DNA in dissected hippocampus and cortex of 6 *SCN8A*-ABE–treated *Scn8a*^W/+^-EIIa mice that survived past P21, a timepoint where all sham control *Scn8a*^W/+^-EIIa mice had succumbed to death (Supplemental Figure 4, B and C). The percentage of reads from all cells in these regions with the variant T/A at the R1872W locus of *SCN8A* was reduced from an average of 93% ± 0.8% (with calculated 7% failure of heterozygous EIIa-Cre activation of the R1872W variant) to 73% ± 1.8%. WT base pairing of C/G at the R1872W locus increased with an equivalent percentage to the decrease in mutant T/A in *SCN8A*-ABE–treated *Scn8a*^W/+^-EIIa mice (Supplemental Figure 4C).

In both *Scn8a*^W/+^-EMX1 and *Scn8a*^W/+^-EIIa mice, *SCN8A*-ABE treatment effectively converted the tryptophan-encoding TGG sequence of the mutant allele at the R1872W locus to its WT arginine-encoding CGG counterpart, thereby inducing increasing levels of healthy WT transcript.

### In vivo RNA and WGS off-target analysis of SCN8A-ABE–treated mice.

**Figure 3:**
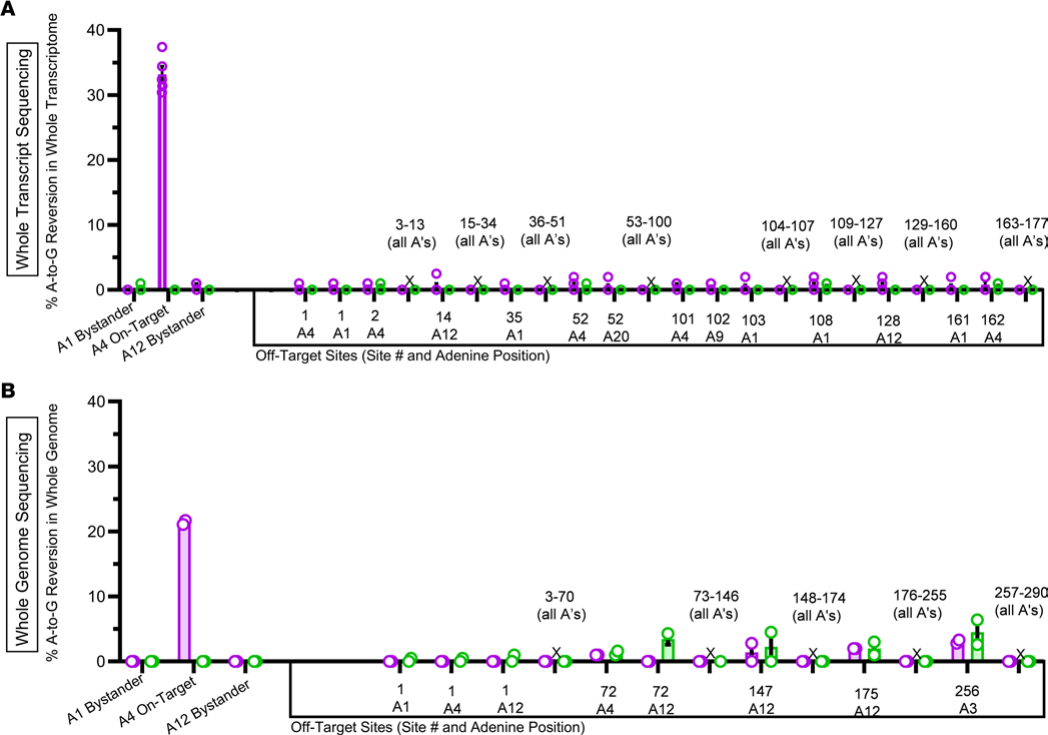
*In vivo* correction of the mutant T in tryptophan codon R1872W. (**A**) On-target R1872W A4 A-to-G percentage reversion activity is compared with bystander adenines A1 and A12 and all adenines at 177 potential off-target exons from *Scn8a*^W/+^-EMX1 *SCN8A*-ABE–treated (*n =* 5, P345) mice compared with *Scn8a*^W/+^-EMX1 sham control (*n =* 5, P25-30) mice. Sites exhibit a less than 5 nt mismatch cutoff from homology to guide and target sequence. X indicates no A-to-G recorded activity at adenines in loci. Data points represent individual mice. (**B**) On-target A4 A-to-G percentage editing results are depicted from WGS in matched pairs of *SCN8A*-ABE treated *Scn8a*^W/+^-EMX1 mice (*n =* 2, P345) and sham control *Scn8a*^W/+^-EMX1 mice (*n =* 2, P25 and P30) compared with bystander adenines A1 and A12 and all adenines at a total 290 potential off-target sites in matched pairs of *SCN8A*-ABE–treated *Scn8a*^W/+^-EMX1 mice and sham-treated *Scn8a*^W/+^-EMX1 mice. Sites exhibit a less than 5 nt mismatch cutoff from homology to guide and target sequence. X indicates no A-to-G percentage recorded activity at adenines in loci. Bars represent mean ± SEM. Threshold for significant levels of editing greater than or equal to 1%. Data points represent individual mice.

To assess the A-to-G off-target activity of *SCN8A*-ABE, we conducted RNA sequencing on brain tissue from *SCN8A*-ABE–treated *Scn8a*^W/+^-EMX1 mice (*n =* 5; P345) and *Scn8a*^W/+^-EMX1 sham control mice (*n =* 5; P25-30). Our objective was to quantify A-to-G editing and transcript reversion events both at the on-target site (A4) and at any other potential off-target locations across the transcriptome. Utilizing the computational tools CRISPOR (UCSC) (25), CRISPR RGEN tools (26), and COSMID (41), we identified all adenines at a total of 290 potential off-target sites across platforms harboring less than 5 nucleotide mismatches with the target sequence or guide RNA. Whole transcriptome RNA sequencing was performed using *SCN8A*-ABE treated *Scn8a*^W/+^-EMX1 and *Scn8a*^W/+^-EMX1 sham control mice. Figure 3A demonstrates A-to-G percentage editing outcomes for all adenines at 177 predicted off-target exon, protein coding sites showing percent A-to-G editing visible as percentage transcript reversions, comparing *SCN8A*-ABE–treated *Scn8a*^W/+^-EMX1 mice with *Scn8a*^W/+^-EMX1 sham control mice. This comparison controls for sequencing noise inherent to the brain transcriptome, which we observed at less than or equal to 1%, consistent with no biologically meaningful off target activity (42–45). We observed less than or equal to 1% average transcript reversion of 2 adenines at Ap3d1 and 1 adenine each at off-target Agpat3, Cables2, and Inst3 loci in the CNS across the *Scn8a*^W/+^-EMX1 *SCN8A*-ABE–treated mice compared with *Scn8a*^W/+^-EMX1 sham control mice. There was no A-to-G base editing at any of the other adenines at the additional assayed potential off-target loci. In comparison, editing of the target A4 adenine resulted in an average of 32% on-target transcript reversion of mutant-to-normal WT transcripts with less than or equal to 1% bystander A1 and A12, consistent with no biologically meaningful bystander activity (Figure 3A). Absence of significant bystander adenines in *Scn8a*^W/+^-EMX1 *SCN8A*-ABE–treated mice was confirmed via NGS amplicon sequencing validations (Supplemental Figure 3). These results indicate high genomic target specificity of *SCN8A*-ABE for the on-target R1872W locus.

To further characterize off-target editing, we performed 30 × short read WGS on matched pairs of *SCN8A*-ABE–treated *Scn8a*^W/+^-EMX1 (*n =* 2; P345) and sham control *Scn8a*^W/+^-EMX1 mice (*n =* 2; P25–P30) (Figure 3B). We evaluated 977 adenines at a total of 290 potential off-target sites harboring less than 5 nucleotide mismatches with the target or guide sequence, including all sites evaluated in the RNA sequencing analysis. WGS analysis revealed no significant off-target reads of A-to-G altered reads from the 2 pairs of *SCN8A*-ABE–treated mice compared with their matched sham controls at all 977 adenines in all 290 potential off-target loci (Figure 3B, ≤ 1%), consistent with no biologically meaningful off-target activity (42–45). In comparison, an average of greater than 21% on-target editing at the R1872W target A4 adenine was observed with no significant bystander A-to-G editing (≤ 1%), at A1 and A12 (Figure 3B). The results of the comprehensive off-target analysis indicate high genomic target specificity of the *SCN8A*-ABE base editing strategy for the on-target R1872W locus. To further support the specificity of our construct for both *SCN8A* and the R1872W mutant allele, we evaluated all members of the *SCNX* gene family at genomic locations most likely to exhibit off-target activity following *SCN8A*-ABE treatment. These evaluations, based on transcriptome-wide RNA-seq and WGS data, confirmed that no detectable A-to-G editing occurred at any of these loci (Supplemental Table 3).

### ABE treatment attenuates pathological neuronal hyperexcitability and persistent sodium current (I_NaP_).

**Figure 4:**
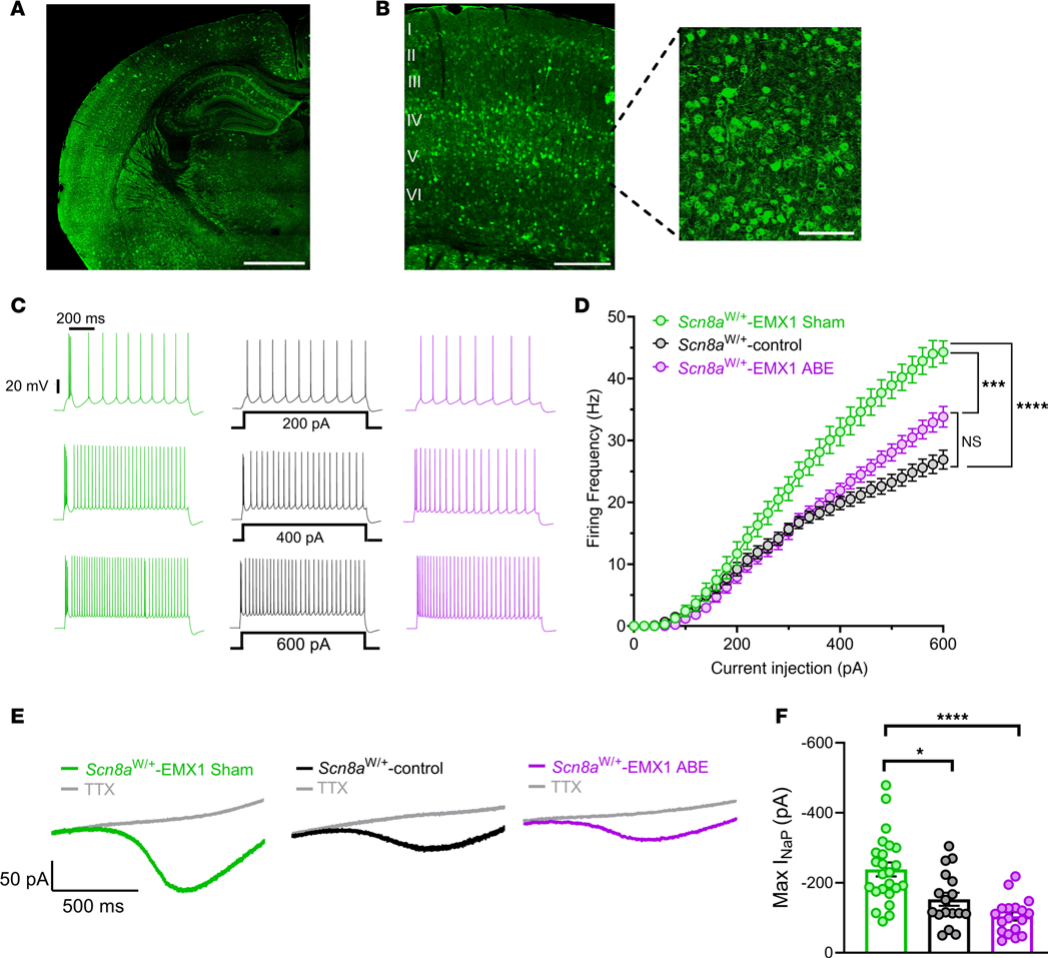
*SCN8A*-ABE attenuates neuronal hyperexcitability and reduces the pathological persistent sodium current (I_NaP_). (**A**) GFP expression in 30 μm sectioned brain tissue (P25) from *Scn8a*^W/+^-EMX1 mouse injected with the *SCN8A*-ABE and AAV-GFP viruses. Scale bar: 0.5 mm. (**B**) GFP labeling of cortical layers (LI-LVI) in *Scn8a*^W/+^-EMX1 mouse injected with *SCN8A*-ABE and AAV-GFP virus (Scale bar 0.2 mm). Inset (Right) shows GFP labeling of cortical layer LIV/LV pyramidal neurons. Scale bar: 0.01 mm. (**C**) Example traces of action potential firing from *Scn8a*^W/+^-EMX1 sham control (green), *Scn8a*^W/+^-control (black), and *Scn8a*^W/+^-EMX1 *SCN8A*-ABE–treated (purple) cortical layer LIV/LV pyramidal neurons at current injection steps of 200 pA, 400 pA, or 600 pA (P17-P25). (**D**) Average number of action potentials elicited relative to current injection magnitude. At current injections greater than 240 pA, *Scn8a*^W/+^-EMX1 Sham neurons (*n =* 25, 4 mice) exhibit increased firing frequencies when compared with *Scn8a*^W/+^-control (*n =* 21, 4 mice). This hyperexcitability is significantly attenuated in *Scn8a*^W/+^-EMX1 *SCN8A*-ABE–treated neurons (*n =* 33 cells, 7 mice; ***P < 0.001, ****P < 0.0001; nested 1-way ANOVA with Dunns’s multiple comparisons test). (**E**) Example traces of steady state I_NaP_ evoked by slow voltage ramps in pyramidal neurons from *Scn8a*^W/+^-EMX1 sham (green), *Scn8a*^W/+^-control (black), and *Scn8a*^W/+^-EMX1 *SCN8A*-ABE–treated mice (purple). Traces in gray show slow voltage ramp in the presence of 500 nM TTX. (**F**) Elevated maximum I_NaP_ in *Scn8a*^W/+^-EMX1 sham control neurons (*n =* 24, 8 mice) is rescued by *SCN8A*-ABE treatment in *Scn8a*^W/+^-EMX1 neurons (*n =* 18, 8 mice) to amplitudes recorded in *Scn8a*^W/+^-control neurons (*n =* 17, 6 mice; *P < 0.05, ****P < 0.0001; Kruskal-Wallis with Dunn’s multiple comparisons). No significant difference observed between *Scn8a*^W/+^ EMX1 *SCN8A*-ABE–treated and *Scn8a*^W/+^-control groups. Data points represent individual recordings, bars represent mean ± SEM.

Increases in neuronal excitability are a hallmark of seizures in *SCN8A* DEE (10, 11), and hyperexcitability of excitatory cortical and hippocampal pyramidal cells was previously reported in the *Scn8a*^W/+^-EIIa mouse model (10). We next determined whether *SCN8A*-ABE treatment could rescue neuronal hyperexcitability (Figure 4). As an additional control, we examined *Scn8a*^W/+^ mice in the absence of Cre, where the R1872W variant is not activated (*Scn8a*^W/+^-control). Electrophysiological recordings were conducted at a time point corresponding to seizure onset via EEG monitoring (Figure 2D). Transduced neurons were identified by GFP expression. Transduction of GFP in *Scn8a*^W/+^-EMX1 mice was widespread, with high staining in the hippocampus and cortex (Figure 4, A and B). We examined neuronal excitability of GFP-positive pyramidal neurons in the somatosensory cortex layer IV/V of P17–25 sham control (*n =* 25, 4 mice) and *SCN8A*-ABE–treated *Scn8a*^W/+^-EMX1 mice (*n =* 33 cells, 7 mice). Sham control *Scn8a*^W/+^-EMX1 neurons were hyperexcitable when compared with *Scn8a*^W/+^-control neurons (*n =* 21, 4 mice), which was attenuated in *SCN8A*-ABE–treated *Scn8a*^W/+^-EMX1 neurons. *SCN8A*-ABE treatment did not fully restore firing frequencies to levels observed in *Scn8a*^W/+^-control mice at higher current injection steps (Figure 4, C and D). Analysis of membrane and action potential (AP) properties showed a significant increase in downstroke velocity in *SCN8A*-ABE–treated *Scn8a*^W/+^-EMX1 neurons compared with sham controls (Supplemental Table 4). All other parameters were unchanged between ABE and sham control *Scn8a*^W/+^-EMX1 neurons.

We also observed heightened neuronal excitability in P13–17 *Scn8a*^W/+^-EIIa sham control mice (*n =* 17, 5 mice) when compared with *Scn8a*^W/+^-control mice (*n =* 29, 5 mice), which was completely rescued by *SCN8A*-ABE treatment (*n =* 26, 8 mice) (Supplemental Figure 5, A and B). Analysis of membrane and action potential properties revealed a decrease in rheobase and downstroke velocity and an increase in AP width in *Scn8a*^W/+^-EIIa sham control mice when compared with *Scn8a*^W/+^-EIIa *SCN8A*-ABE mice. We also observed a decrease in AP threshold in both sham control and *SCN8A*-ABE–treated *Scn8a*^W/+^-EIIa mice when compared with *Scn8a*^W/+^-controls. (Supplemental Table 5).

The most prominent biophysical feature of *SCN8A* GOF variants is an elevated persistent sodium current (I_NaP_) (3, 11, 13). Elevated I_NaP_ is a major contributor to neuronal hyperexcitability in epilepsy (13). Thus, we sought to determine whether *SCN8A*-ABE treatment eliminated the pathological heightened I_NaP_. We used slow voltage ramps in pyramidal neurons from layer IV/V of the somatosensory cortex to measure I_NaP_ in sham control (*n =* 24; 8 mice) and *SCN8A*-ABE–treated *Scn8a*^W/+^-EMX1 mice (*n =* 18; 8 mice) and compared them with *Scn8a*^W/+^-control mice (P17-P25: *n =* 17; 6 mice). ABE treatment significantly decreased the amplitude of I_NaP_ compared with sham control *Scn8a*^W/+^-EMX1 mice to levels recorded in *Scn8a*^W/+^-control neurons (Figure 4, E and F). Half maximal activation voltages (V_1/2_) were not different between the 3 groups (Supplemental Table 4).

### ABE treatment improves behavioral comorbidities in R1872W SCN8A DEE mice.

**Figure 5:**
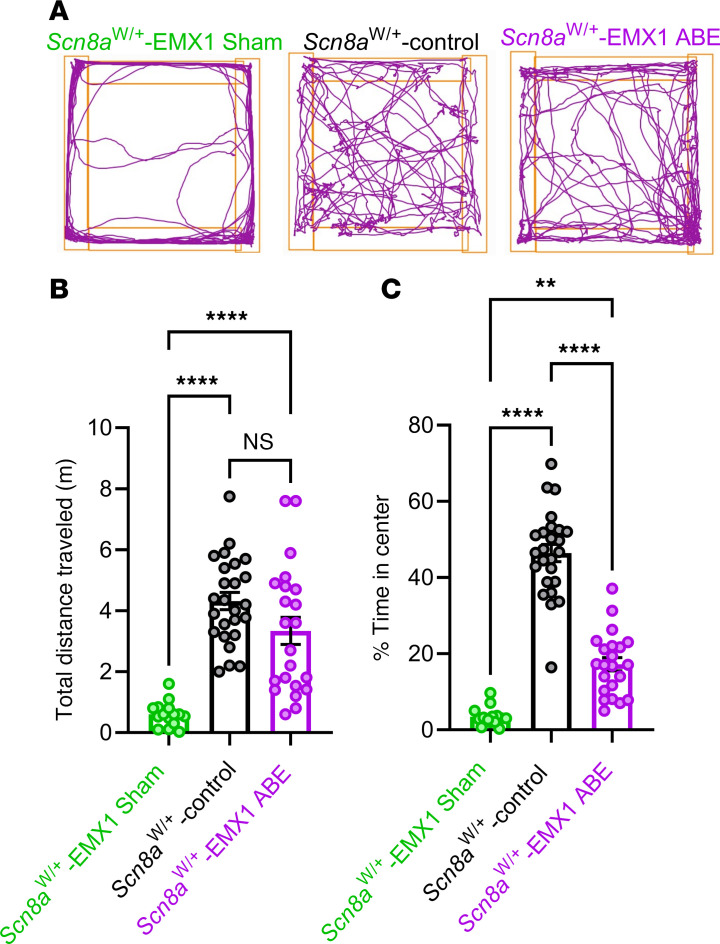
Anxiety and movement discrepancy phenotypes are reduced by *SCN8A*-ABE treatment. (**A**) Representative movement tracks in open field for *Scn8a*^W/+^-EMX1 sham control (*n =* 15), *Scn8a*^W/+^-control (*n =* 25), and *Scn8a*^W/+^-EMX1 *SCN8A*-ABE treated mice (*n =* 22). (**B**) In the open field test, *Scn8a*^W/+^-EMX1 sham control mice (*n =* 15, green) show significantly decreased distance traveled compared with *Scn8a*^W/+^-control mice (*n =* 25, black). Treatment with *SCN8A*-ABE in *Scn8a*^W/+^-EMX1 mice (*n =* 22, purple) rescues this phenotype to levels seen in *Scn8a*^W/+^-control mice (****P < 0.0001, Brown-Forsythe ANOVA with Dunnett’s multiple comparisons test). (**C**) *Scn8a*^W/+^-EMX1 sham control mice (*n =* 15, green) show significantly reduced time in the center in the open field test compared to *Scn8a*^W/+^-control mice (*n =* 25, black). *Scn8a*^W/+^-EMX1 *SCN8A*-ABE treatment attenuated this phenotype (*n =* 22, purple) but did not reach *Scn8a*^W/+^-control levels (**P < 0.01, ****P < 0.0001, Kruskal-Wallis test with Dunn’s multiple comparisons test). Data points represent individual mice, bars represent mean ± SEM.

In addition to seizures, patients with *SCN8A* DEE suffer from severe comorbidities, including movement disorders and cognitive impairment (5). To examine locomotor activity and anxiety-like behavior, we evaluated both sham control (*n =* 15) and *Scn8a*-ABE–treated (*n =* 22) *Scn8a*^W/+^-EMX1 mice along with *Scn8a*^W/+^-control mice (*n =* 25) in the open field test. We used total distance travelled to assess locomotor activity and thigmotaxis as a measure of anxiety-like behavior (46). Mice were tested between 4 and 8 weeks of age, a time point after the onset of seizures in *Scn8a*^W/+^-EMX1 mice (10). We generated quantifications of locomotor activity to analyze behavior in all groups (Figure 5). *Scn8a*^W/+^-EMX1 sham control mice had reduced locomotor activity and spent significantly less time in the open areas of the field compared with *Scn8a*^W/+^-control mice, indicating diminished mobility and increased anxiety (Figure 5, A–C). Treatment with *SCN8A*-ABE significantly improved both comorbidities, with locomotor activity completely restored to levels observed in *Scn8a*^W/+^-control mice (Figure 5B). Thigmotaxis was significantly reduced in *SCN8A*-ABE–treated *Scn8a*^W/+^-EMX1 mice but did not reach *Scn8a*^W/+^-control levels (Figure 5, A and C). We also tested general cognition and memory. We observed no significant differences in recognition memory (more time in the novel arm), spatial reference memory (more visits to the novel arm), or working memory (spontaneous alterations recorded) between the groups (Supplemental Figure 6). These findings collectively suggest that *Scn8a*^W/+^-EMX1 mice have underlying motor discrepancies and anxiety that can be ameliorated by early *SCN8A*-ABE treatment.

## Discussion

Treatments for severe genetic epilepsy disorders are limited, and current therapeutic approaches do not target the underlying mechanisms. Here, we provide compelling evidence that base editors can serve as an effective and targeted therapeutic approach for one *SCN8A* DEE variant, R1872W, validating the potential for targeted treatment of other *SCN8A*-related DEE variants.

Our results show that *SCN8A*-ABE treatment successfully reduced seizure burden of mice carrying the *SCN8A* R1872W variant, which increased survival and improved associated behavioral comorbidities. Neuronal hyperexcitability, a hallmark of seizure activity, and elevation of persistent sodium current (I_NaP_) associated with some *SCN8A* GOF variants (11, 13) were also attenuated in *SCN8A*-ABE–treated mice. These findings support a reversal of the pathogenic GOF physiology of the R1872W variant via *SCN8A*-ABE treatment. This substantial phenotypic rescue was achieved by correction of DNA that resulted in approximately 32% absolute reversion of the mutant *SCN8A* transcripts, which is primarily expressed by neurons (2, 47). RNA and WGS revealed no significant off-target or bystander adenine effects, supporting high specificity of editing of the *SCN8A*-ABE therapy for the R1872W mutant allele. The use of an optimized guide RNA along with a highly specific evolved ABE8e base editor minimized off-target effects while preserving high editing efficiency. These findings underscore the transformative potential of precise gene-editing interventions in genetic epilepsy disorders where current pharmacological treatments remain ineffective.

Here, we demonstrate the potential of base editing to treat a genetic form of epilepsy, but other gene therapies have been explored previously as a treatment for *SCN8A* DEE, Dravet Syndrome, and other severe genetic epilepsy syndromes. Antisense oligonucleotides (ASOs) ameliorate many aspects of the DEE phenotype in mice (48–52), and approximately 25% reduction of *SCN8A* transcripts has been shown to rescue the premature death phenotype of *SCN8A* DEE (49). However, ASOs require continuous administration due to turnover within a few weeks to months of injection (53). Furthermore, the turnover rate of Na_v_1.6 transcripts, which is not completely understood (1), could also impact the success of ASOs in *SCN8A* DEE (54). In contrast, base editing can offer permanent treatment to revert pathogenicity by directly targeting the genetic cause of the *SCN8A* DEE phenotype. In this study, we achieved a 32% permanent reversion of mutant to WT transcripts, indicating that our editing efficiency is more than sufficient for phenotypic rescue. Allele-specific editing using CRISPR/Cas9 has been explored as a therapeutic strategy for *SCN8A* DEE in mice and demonstrated the feasibility of specifically inactivating the pathogenic *SCN8A* variant (55). By using silent mutations to create sequence differences between mutant and WT alleles, selective targeting of the mutant allele was achieved. However, while this strategy was effective in mice engineered to ubiquitously express Cas9, translating it to human patients is more complex due to the absence of natural sequence variation and challenges in Cas9 delivery. Nonetheless, this study provides a critical foundation, demonstrating that CRISPR-based modifications of sodium channel genes can drive functional changes, guiding future therapeutic endeavors. In addition to gene therapy efforts, small molecules that selectively target the Na_v_1.6 sodium channel isoform could provide an additional tool, in combination with gene therapy approaches, to achieve complete seizure freedom in these patients (56).

The most striking effect of *SCN8A*-ABE treatment was the complete inhibition of seizure activity observed in the majority of *SCN8A*-ABE–treated *Scn8a*^W/+^-EMX1 mice and a significant reduction in seizure frequency and severity in the remaining mice. This directly translated to increased survival in *Scn8a*^W/+^-EMX1 mice and likely the same for the *SCN8A*-ABE–treated *Scn8a*^W/+^-EIIa mice. In addition to seizures, patients with *SCN8A* DEE experience severe comorbidities, including diminished mobility and anxiety-like behaviors (57, 58). Our behavioral analyses revealed that *SCN8A*-ABE treatment significantly improved locomotor activity and reduced anxiety-like behaviors in *Scn8a*^W/+^-EMX1 mice. This suggests that restoring early Na_v_1.6 function may have therapeutic benefits beyond seizure suppression, addressing other debilitating aspects of the disorder.

The physiological consequences of *SCN8A* GOF variants include an elevated I_NaP_ due to disrupted channel inactivation and subsequent neuronal hyperexcitability (3, 11, 10). Consequentially, many pharmacological treatments for *SCN8A* DEE target I_NaP_ directly (59). *SCN8A-*ABE treatment ameliorated the pathological increase in I_NaP_ in cortical neurons to nonpathological levels recorded in control mice, which likely led to the rescue of neuronal excitability in *SCN8A*-ABE–treated cortical neurons. Expression of the *SCN8A* R1872W variant selectively in cortical neurons is sufficient for seizure onset and seizure-induced death (10). It remains to be determined if selective targeting in a young adolescent model of a global *SCN8A* variant–expressing mouse model would be sufficient for seizure suppression.

A major concern in gene-editing applications is the potential for off-target modifications that could result in unintended consequences. RNA transcriptome-wide and WGS analysis revealed high specificity of *SCN8A*-ABE, with no significant DNA or RNA off-target or bystander adenine A-to-G conversion detected. This is likely due to the high specificity of the guide RNA as well as the use of ABE8e (V106W) -NRCH. ABE8e itself demonstrates a minor degree of off-target editing, which was ameliorated with the addition of variant V106W in the TadA deaminase domain of ABE8e (19). We elected to induce the V106W variant via site directed mutagenesis (SDM) into ABE8e-NRCH to achieve off-target reductions. Studies have demonstrated that adenine base editors have the ability to cause off-target effects within the genome (60). Unintended modifications at nontarget sites pose significant challenges for clinical applications of all genome editors. These unintended mutations can lead to disruption of essential genes or the activation of oncogenes, potentially resulting in severe side effects like tumor development (61). To mitigate these risks, it is crucial to develop strategies like the V106W variant incorporation and highly specific guide RNAs that enhance the precision of base editors.

DNA A-to-G editing levels at or below approximately 13% were consistently associated with premature mortality (< P150) in the *SCN8A*-ABE–treated *Scn8a*^W/+^-EMX1 mice. Conversely, achieving editing efficiencies above 14% resulted in a significant amelioration of premature death, though recurrent seizures were still observed in some mice. Complete seizure cessation and the prevention of seizure-induced death were exclusively observed in mice that achieved editing efficiencies over 17%. Similarly, percentage of A-to-G editing efficiencies of 15% and above resulted in a significant amelioration of premature mortality in the *SCN8A*-ABE–treated *Scn8a*^W/+^ -EIIa mice (≥ P21).

We observed higher on-target RNA transcript levels of reversion (approximately 32%) of the mutant expression than observable in DNA (approximately 19%) following the delivery of the AAV vector PhP.eB. This observation reflects the preferential transfection of neurons by PhP.eB, highlighting key aspects of cell-type–specific editing dynamics. It also demonstrates the phenomenon of *SCN8A* gene being preferentially expressed in neurons in the CNS; we observed more limited DNA editing as many glial subtypes and other cells in the brain do not express *SCN8A* (2, 47).

We used *Scn8a*^W/+^-EMX1, a model that allows expression of the R1872W variant selectively within forebrain excitatory neurons, to overcome the early onset of seizure-induced death as a result of global expression of the variant, as recorded in *Scn8a*^W/+^ -Ella mice. A limitation to using the EMX1 mouse model is that the variant is expressed exclusively in excitatory neurons of the forebrain. This excludes several types of interneurons known to be affected by expression of the *SCN8A* variant, including parvalbumin-positive (PV-positive), γ-aminobutyric acid–positive (GABAergic) neurons (62), somatostatin (SST) interneurons (63), and reticular thalamus neurons (64). The role of interneurons in the context of gene editing remains to be determined.

While this study provides strong evidence for the therapeutic potential of base editing in *SCN8A* DEE, several challenges remain. The observed editing efficiency, while sufficient for phenotypic rescue, remains below the theoretical maximum. Future strategies to enhance editing, such as optimizing AAV dosing with dual guide RNA exposure (31) or employing novel viral capsids or alternative delivery methods, such as lipid nanoparticles with improved CNS-wide targeting, may be required for full symptomatic relief in these patients. Since patients with *SCN8A* DEE typically exhibit seizure onset at approximately 4–6 months of age and can be identified shortly after that time (65), a major consideration for the applicability of base editing in these patients is the time of delivery. In our study, both *Scn8a*^W/+^-EIIa and *Scn8a*^W/+^-EMX1 mice were treated at postnatal day 2, a timepoint before the onset of spontaneous seizures. In human patients, ABE treatment would be initiated after seizure onset. This is true for other genetic epilepsy syndromes as well (48, 49, 52, 55). Administration of a *SCN8A* ASO or shRNA after seizure onset has been shown to be protective against SUDEP in 2 models of *SCN8A* DEE (51). While future studies are needed to assess the efficacy of base editing in *SCN8A* DEE after seizure onset, it is likely that a “therapeutic window” can be identified to allow AAV expression and turnover of the pathologic protein.

Patients with variants at residue 1872 constitute approximately 10%–15% of reported cases of *SCN8A* DEE (5, 6, 8). Many other recurrent GOF *SCN8A* variants such as R1617Q lead to a DEE phenotype (66), and these could be targeted by base editors for precise therapeutic interventions that address the underlying genetic cause of the disease. Base editing technology may also be utilized to target severe single-nucleotide variants responsible for other genetic epilepsy syndromes. As gene-editing technologies continue to advance, base editing approaches such as *SCN8A*-ABE pave the way for future clinical applications of gene therapies in genetic forms of epilepsy.

## Methods

### Sex as a biological variable.

Both sexes experience *SCN8A* DEEs with equivalent proportions, there is no known sex-linked interaction in *SCN8A* primary function. Both male and female mice were used for this study with no discrimination to sex.

### Cell culture.

Details regarding cell culture protocol and conditions (67, 68) can be found in Supplemental Material.

### Cloning and viral packaging.

Details regarding plasmids used in this study and subsequent cloning and viral packaging can be found in Supplemental Material.

### Base editor construct characterization.

For characterization of base editing constructs, mutation-integrated cells were seeded 16–18 h before transfection. Both cell types (HEK293T-R1872W and CHO-R1872W) were subjected to transfection at approximately 70% confluency with a plasmid encoding the base editor and a separate plasmid harboring the sgRNA-P2A-GFP construct. Transfection of base editor and guide construct occurred at a 3:1 mass ratio using Lipofectamine 3000 (ThermoFisher Scientific) as previously described (19) and in accordance with the manufacturer’s protocols, but scaled up into a 6-well plate (Corning). This scale-up was done to evaluate high cell density conditions in either cell line. Cells did not undergo antibiotic selection or GFP cell sorting after base editor exposure during transfection and were cultured for 72 hours before harvesting. We collected gDNA from cells 72 hours after transfection and Genomic DNA was isolated using PurelinkDNA mini Kit (Invitrogen). PCR conditions and primers are detailed in Supplemental Material. PureLink PCR Purification Kit was utilized following completion of PCR. Sanger sequencing of the purified product of edited *SCN8A* in HEK293T-R1872W and CHO-R1872W cells was performed at Eurofins. Genomics and analysis of files were conducted with EditR1.0.10 as previously described (69) and verified on SnapGene v.10.0.3.

### Mouse husbandry and genotyping.

Husbandry details can be found in Supplemental Material. Homozygous Cre females were used for breeding to ensure minimal germline recombination due to Cre, as shown previously (70, 71). For all experiments, we used *Scn8a*^W/+^ mice as controls that contained the same allele encoding the *SCN8A* variant but lacked Cre activation and therefore only expressed WT Na_v_1.6 from both alleles (32).

### Viral vectors and intracerebroventricular injections.

AAV vector backbone plasmids were cloned to insert the sgRNA sequence and C-terminal base editor half of ABE8e-NRCH (V106W) into v5 Cbh-AAV-ABE-NpuC+U6-sgRNA (Addgene 137177), and the N-terminal base editor half v5 Cbh-AAV-ABE-NpuN (Addgene 137178) (Genscript Inc.) (31). Viral capsid preparations, packaging, and purification of PhP.eB -AAV–packaged base editor plasmids were performed by Salk Institute’s Gene Transfer, Targeting and Therapeutics Viral Vector Core. High-titer qualified AAV was stored at –80°C until use. Neonatal ICV injections were performed as previously described (10). *Scn8a*^W/+^-EIIa and *Scn8a*^W/+^-EMX1 mice were given a single unilateral ICV (2 μL volume) of either 2.0 × 10^10^ viral genomes (vg) PhP.eB-CMV-GFP in PBS (Sham) or 1.0 × 10^11^ vg of the *SCN8A*-ABE treatment in PBS (5.0 × 10^10^ vg of each dual intein PhP.eB-ABE vectors) along with 2.0 × 10^10^ vg PhP.eB-GFP at P1 using a 33-gauge needle attached to a 5 μL microvolume syringe as previously described (72). This dosage aligns with high therapeutic doses used for P0 ICV AAV administration of base editors for rescuing Hutchinson-Gilford Progeria syndrome and Niemann-Pick disease in mice (31, 73).

### Whole brain immunofluorescence imaging.

Brain tissue for immunohistochemistry was processed as follows: Mice were anesthetized and transcardially perfused with 10 ml ice-cold Dulbecco’s PBS (DPBS, Gibco, 14200-075) followed by 10 ml ice-cold 4% PFA. Brains were fixed in 4% paraformaldehyde (PFA) and 30 μm coronal brain sections were obtained using a cryostat. Floating sections were fixed and stored in DPBS. Sections were permeabilized with 0.1% Triton X-100 in DPBS for 30 minutes and blocked with 2% bovine serum albumin (BSA) for 2 hours. Sections were incubated with the primary antibody, polyclonal rabbit anti-GFP (Abcam, ab290), diluted in DPBS containing 2% BSA at a concentration of 1:1000. The secondary antibody, AlexaFluor-488 goat anti-rabbit (A11034; Invitrogen), was diluted 1:500 in DPBS containing 2% BSA. Sections were stained free-floating in primary antibody on a shaker at 4°C overnight and with secondary antibody for 2 h at room temperature the following day. Sections were mounted using Prolong Gold antifade reagent with DAPI (Invitrogen, P36935) on a microscope slide. Images were gathered using Zeiss Zen software on a LSM700 or LSM880. Whole brain images were stitched following acquisition with a 20× objective (Plan-Apochromat 20 ×/0.8) and z-stack images were gained by use of a 63x objective (Plan-Apochromat 63 ×/1.4).

### Brain slice preparation.

Preparation of acute brain slices for patch-clamp electrophysiology experiments was modified sparingly from standard protocols previously described (11). Mice were anesthetized with isoflurane and decapitated. The brains were rapidly removed and kept in chilled slicing solution (1°C) containing (in mM): 93 N-Methyl-D-glutamine (NMDG), 2.5 KCl, 1.25 NaH_2_PO_4_, 20 HEPES, 5 L-ascorbic acid (sodium salt), 2 thiourea, 3 sodium pyruvate, 0.5 CaCl_2_, 10 MgSO_4_, 25 D-glucose, 12 N-acetyl-L-cysteine, 30 NaHCO_3_; pH adjusted to 7.4 using HCl (osmolarity 310 mOsm). Slices were continuously oxygenated with 95% O_2_ and 5% CO_2_ throughout the preparation. 300 μm coronal brain sections were prepared using a Leica Microsystems VT1200 vibratome. Slices were collected and placed in warmed (37°C) ACSF containing (in mM): 125 NaCl, 2.5 KCl, 1.25 NaH_2_PO_4_, 2 CaCl_2_, 1 MgCl_2_, 0.5 L-ascorbic acid, 10 glucose, 25 NaHCO_3_, and 2 Na-pyruvate. Slices were incubated for approximately 30 min and then kept at room temperature for up to 6 h.

### Intrinsic excitability recordings.

Brain slices were placed in a chamber superfused (2 ml/min) with continuously oxygenated recording solution warmed to 32 ± 1°C. Whole-cell recordings were performed using a Multiclamp 700B amplifier with signals digitized by a Digidata 1550B digitizer. Currents were amplified, lowpass filtered at 2 kHz, and sampled at 100 kHz. Borosilicate electrodes were fabricated using a Brown-Flaming puller (model P1000, Sutter Instruments) to have pipette resistances between 3 and 5 mΩ. Current-clamp recordings of neuronal excitability were collected in ACSF solution. The internal solution contained the following (in mM): 120 K-gluconate, 10 NaCl, 2 MgCl_2_, 0.5 K_2_EGTA, 10 HEPES, 4 Na_2_ATP, 0.3 NaGTP, pH 7.2 (osmolarity 290 mOsm). Intrinsic excitability was assessed using methods adapted from those previously described (11). Resting membrane potential was manually recorded from the neuron at rest. The first AP at rheobase, defined as the maximum amount of depolarizing current that could be injected into neurons before eliciting an AP, was used to calculate passive membrane and AP properties, including threshold; upstroke and downstroke velocity, which are the maximum and minimum slopes on the AP, respectively; amplitude, which was defined as the voltage range between AP peak and threshold; APD_50_, which is the duration of the AP at the midpoint between threshold and peak; and input resistance, which was calculated using a −20 pA pulse in current-clamp recordings. AP frequency–current relationships were determined using 1 s current injections from −140 to 600 pA. All patch-clamp electrophysiology data were analyzed using custom MATLAB R24.a scripts and/or ClampFit 11.2.

### Persistent sodium current (I_NaP_) recordings.

The recording solution has been previously described (11) and contained (in mM): 100 NaCl, 40 TEACl, 10 HEPES, 3.5 KCl, 2 CaCl_2_, 2 MgCl_2_, 0.2 CdCl_2_, 4 4-aminopyridine (4-AP), 25 D-glucose. The internal solution for sodium channel recordings contained the following (in mM): 140 CsF, 2 MgCl_2_, 1 EGTA, 10 HEPES, 4 Na_2_ATP, and 0.3 NaGTP with the pH adjusted to 7.3 and osmolality to 300 mOsm. Steady-state persistent sodium currents (I_NaP_) were elicited using a voltage ramp (20 mV/s) from −80 to −20 mV. After collecting recordings at baseline, protocols were repeated in the presence of 500 nM tetrodotoxin (TTX; Alomone Labs) to completely isolate I_NaP_. TTX-subtracted traces were analyzed by subtracting the current recorded in the presence of TTX from the current recorded in its absence. The half-maximal voltage for activation was calculated as previously described (74).

### EEG Recordings.

Custom electroencephalogram (EEG) headsets (Digikey) were implanted in P18 *Scn8a*^W/+^-EMX1 mice using standard surgical techniques as previously described (75). Anesthesia was induced with 5% and maintained with 0.5%–3% isoflurane. Adequacy of anesthesia was assessed by lack of toe-pinch reflex. A midline skin incision was made over the skull and connective tissue was removed. Burr holes were made at the lateral/anterior end of the left and right parietal bones to place EEG leads and at the interparietal bone for ground electrodes. EEG leads were placed bilaterally in the somatosensory cortex and unilaterally placed in the occipital lobe. A headset was attached to the skull with dental acrylic (Jet Acrylic; Lang Dental). Mice received postoperative analgesia with ketoprofen (5 mg/kg, i.p.) and 0.9% saline (0.5 mL i.p.) and were allowed to recover a minimum of 2–4 days before seizure-monitoring experiments. Mice were then individually housed in custom-fabricated chambers and monitored for the duration of the experiment. The headsets were attached to a custom low-torque swivel cable, allowing mice to move freely in the chamber. EEG signals were amplified at 2000× and bandpass filtered between 0.3 and 100 Hz, with an analog amplifier (Neurodata model 12, Grass Instruments). Biosignals were digitized with a Powerlab 16/35 and recorded using LabChart 7 software at 1 kS/s. Video acquisition was performed by multiplexing four miniature night vision-enabled cameras and then digitizing the video feed with a Dazzle Video Capture Device and recording at 30 fps with LabChart 7 software in tandem with Biosignals. Modified Racine Scale was used to determine seizure severity in each epileptic indication. Behavioral responses were scored as: 0, normal behavior; 1, behavioral arrest; 2, orofacial automatisms; 3, unilateral forelimb clonus; 4, bilateral forelimb clonus and rearing; and 5, rearing with loss of balance (76, 77).

### Next generation amplicon genomic DNA sequencing.

Sequencing library preparation was performed according to previously published protocols (24). We isolated genomic DNA (gDNA) with the Invitrogen PureLink Genomic DNA Mini Kit and approximately 25 mg of homogenized tissue was used for individual locus editing experiments. DNA from hippocampal and cortical tissue was microdissected and homogenized for genomic processing. Sequencing libraries were amplified in 3 steps, first to amplify the mutated locus of interest to confirm editing of the mutant allele without healthy read noise from the WT allele of the heterozygote mice (FP1/RP1). This was accomplished by detection of TALEN-induced silent mutations in exons 26b and 26a, which are placed into the mutant R1872W allele only and are not present in the WT allele (10, 32, 41). The second primer set functioned to shorten the length of the amplicon to 150 bp (FP2/RP2); and third to add full-length Illumina sequencing adapters. PCR conditions and adapter primers are detailed in Supplemental Material. All subsequent library preparations and Illumina 2×150 bp sequencing was performed by Azenta (Genewiz) Inc. The samples were sequenced via alignment of fastq files and quantification of allele frequency for individual loci were performed using CRISPResso2 in batch mode (78). The allele frequency for each site was calculated as the number of reads containing the full guide site along with nucleotides C/G at the R1872W locus and the total number of variants reads containing nucleotides T/A at the R1872W locus in the application. Default base editing parameters were used for both experimental sham control and *SCN8A*-ABE groups on batch mode in CRISPResso2 to measure substitution presence in guide sequence of the amplicon in sham control and *SCN8A*-ABE mice. Default parameters and direct comparisons of experimental groups were used to detect the equivalent present noise and nonfull alignments to the target sequence.

### Whole-transcriptome RNA seq.

5 *Scn8a*^W/+^-EMX1 sham control and a total 13 *Scn8a*^W/+^-EMX1 *SCN8A*-ABE treated mice (P345) were taken down; hippocampal and cortical tissues were immediately dissected and collected for evaluation of RNA transcripts. Total RNA was harvested from cells in the hippocampus and cortex using the RNeasy Mini kit (Qiagen). Library preparation and sequencing were performed by Azenta “Genewiz”. Sequencing and subsequent FASTQs were performed and generated by Azenta “Genewiz” in paired-end mode on Illumina Novaseq. FastQC (v0.11.5) was used to assess the quality of each sample. Cutadapt (v3.4) was used to trim adapters. QC controlled FATSQ reads were then converted to BAM/BAI sequencing files via SAMTools and aligned to the mm39 murine reference genome. Mice (*n =* 5) from each group’s trimmed reads were then aligned to the GENCODE mouse reference genomeGRCm39 using IGV genome analysis browser and refined to canonical coding sequences using CCDS release 21 (79) to confirm the absence of editing at all adenines at 177 potential off-target sites harboring less than 5 mismatches with the target sequence or guide RNA within coding regions of DNA identified with CRISPOR (UCSC) (25), CRISPR RGEN tools (26), and COSMID (41). Default parameters were used to remove low-quality bases, adapter sequences, low reads counts (total target reads ≤ 3, and variant reads ≤ 1 in 1 out of 10 experimental mice), and unpaired sequences (42–45). 5 *Scn8a*^W/+^-EMX1 sham control and 5 *Scn8a*^W/+^-EMX1 *SCN8A*-ABE–treated mice (P345) were compared at all adenines. Expression of the mutant and WT alleles in heterozygous *Scn8a*^W/+^-EMX1 mice confirmed equal allelic expression of both sham control and *SCN8A*-ABE–treated groups. This was accomplished by comparing full WT transcripts to the detection of TALEN-induced silent mutations in exons 26b and 26a (10, 32, 40) on the same platforms as described above.

### Whole-Genome Sequencing.

2 sets of paired *Scn8a*^W/+^-EMX1 sham control and *SCN8A*-ABE–treated mice were euthanized; hippocampal and cortical tissues were immediately dissected and collected for evaluation of WGS. Total DNA was harvested from cells in the hippocampus and cortex using the Purelink Genomic DNA Mini Kit (Invitrogen). Library preparation and sequencing were performed by Azenta “Genewiz”. Sequencing and subsequent FASTQs were performed and generated by Azenta “Genewiz” on Illumina MiSeq. FastQC (v0.11.5) was used to assess the quality of each sample. Cutadapt (v3.4) was used to trim adapters. QC controlled fastq reads were then converted to BAM/BAI sequencing files via SAMTools and aligned to the GENCODE mouse reference genomeGRCm38/mm10 using IGV genome analysis browser to confirm the absence of editing at all adenines at a total 290 potential off-target sites harboring less than 5 mismatches with the target sequence or guide RNA identified with CRISPOR (UCSC) (25), CRISPR RGEN tools (26), and COSMID (41). Default parameters were used to remove low-quality bases, adapter sequences, low reads counts (variant reads ≤1 in 1 out of 4 experimental mice) and unpaired sequences (42–45).

### Behavioral assay: open field test.

All behavioral tests were conducted in behavioral testing rooms between 10:00 and 14:00 hours of the day during the light phase of the light/dark cycle. Behavioral tests were performed in mice between the ages of 4 and 8 weeks. Mice were tested in a random order. After the tests, the equipment was cleaned with 70% ethanol to eliminate olfactory cues. Exploratory behavior, anxiety-like behavior, and general locomotor activity were examined using the open-field test according to previous studies (46). Each mouse was placed in the center of the apparatus consisting of a square area surrounded by white acrylic walls (45 × 45 × 40 cm). The total distance travelled (m) and time spent in the central area (s) were recorded. The central area was defined as the middle 20 × 20 cm area of the field. Data was collected over a 5-min period using the ANY-MAZE software. Data analysis was performed in Microsoft Excel. All statistical comparisons were made using the appropriate test in GraphPad Prism 10.

### Behavioral assay: Y-maze.

Spatial working memory was assessed using a white acrylic Y-maze apparatus (arm length: 40 cm, arm bottom width: 3 cm, arm upper width: 10 cm, wall height: 12 cm). Mice were placed at the center of the Y-maze for 5 min. Mice were tested with no previous exposure or habituation to the maze. A spontaneous alternation was defined as an entry into three different arms on consecutive choices. The percentage of alternation was calculated as the ratio of actual to maximum number of alternations. Time in novel arm, novel arm entries, and number of alternations were recorded and analyzed using the ANY-MAZE software exported to Excel.

### Statistics.

Survival analysis was compared by Mantel-Cox comparison test. Seizure incidence was compared by Mann-Whitney comparisons test. Comparisons of 2 groups, namely genomic data, were compared by an unpaired *t* test when the data were normally distributed with equal variances, by Welch’s *t* test (2-tailed) when the data were normally distributed with unequal variances, and by Mann-Whitney test when the data were not normally distributed. Comparisons of 3 groups, namely, membrane and AP properties, peak sodium currents, and behavioral studies, were compared by 1-way ANOVA followed by Dunnett’s multiple comparisons test when the data were normally distributed with equal variances, by Brown-Forsythe ANOVA with Dunnett’s multiple comparisons test when the data were normally distributed with unequal variances, and by the nonparametric Kruskal-Wallis test followed by Dunn’s multiple comparisons test when the data were not normally distributed. Data were assessed for normality using the Shapiro-Wilk test. Data were tested for outliers using the ROUT method to identify outliers. A nested 1-way ANOVA followed by Tukey’s test with multiple comparisons was used to compare groups in experiments in which repetitive measures were made from a single cell over various voltage commands or current injections. Data are presented as individual data points and/or mean ± SEM. Exact *n* and *P* values are reported in figure legends; *P* values less than 0.05 were considered statistically significant.

### Study approval.

All experiments using live animals were approved by the University of Virginia Animal Care and Use Committees.

### Data availability.

All Supporting data values of each figure can be accessed in Supporting Data Values file. Plasmids are obtainable by request of corresponding authors. All NGS and high-throughput sequencing data has been deposited in the National Institute of Health Sequence Read Archive (SRA) under submission: https://www.ncbi.nlm.nih.gov/sra/PRJNA1356903

## Author contributions

CMR and MKP conceptualized the study. CMR, ARB, TCJD, MPP, SK, and MSY acquired data for the study. CMR, ARB, TCJD, RMM, AMLB, and EJF analyzed data for the study. CMR and RMM compiled all figures for the manuscript. CMR drafted the manuscript. CMR, RMM, MHM, CRF, and MKP edited the manuscript.

## Funding Support

This work is the result of NIH funding, in whole or in part, and is subject to the NIH Public Access Policy. Through acceptance of this federal funding, the NIH has been given a right to make the work publicly available in PubMed Central.

- NIH grants NS103090, NS122834, and NS120702 to MKP; NS34509 and GM24872 to MHM and F31 NS134264 to RMM.
- UVA Brain Institute and Strategic Investment Fund Presidential Fellowship to CMR.
- UVA Brain Institute and Strategic Investment Fund Transformative Neuroscience Pilot Grant to MKP and CRF.
- Ivy Biomedical Innovation Fund Award to MKP.

## Supplementary Material

Supplemental data

Supplemental video 1

Supporting data values

## Version 1 02/02/2026

Electronic publication
