# Supplementary material for: Base editing rescues seizures and sudden death in a SCN8A mutation-associated developmental epileptic encephalopathy model
Source: J Clin Invest. 2026 Feb 2;136(3):e196402. doi: 10.1172/JCI196402 (PMC12871382; doi:10.1172/JCI196402)
Supplement: Supplemental data [file jci-136-196402-s341.pdf]

Base Editing Rescues Seizures and Sudden Death in a *SCN8A* Mutation-Associated Developmental Epileptic Encephalopathy Model.

Supplemental Material

Cell Culture

HEK293 cells with heterozygous expression of the R1872W variant were generated by the UVA Genome Engineering Shared Resource (GESR) Core. A 34-base-long double-stranded FRT sequence was introduced into the AVS1 AAV integration locus on chromosome 19 of HEK293 cells (ATCC) with CRISPR-Cas9. For stable integration of plasmids, these stably expressing FRT-HEK293 cells were co-transfected with two plasmids at an equimolar ratio as previously described (67): the first being pCAG-Flp recombinase (Addgene Plasmid #13787) and the second a plasmid containing a single loci of FLP sites flanking the *SCN8A* coding sequence with the R1872W variant (8,68). The latter plasmid, prior to transfection, was subjected to SDM to include the R1872W variant as well as three silent mutations (10,32,40) that were introduced to allow for polymerase chain reaction (PCR) amplification, accomplished by primer pair FP1 and RP1, of the mutant allele only. This approach streamlined workflow and improved accuracy of on-target analysis. Consequently, the transfection mentioned above allowed for the insertion of the mouse-codon-optimized synthetic *SCN8A* sequence into the AVS1 locus in a single loci on one chromosome of the HEK293 chr.19 triploid group via FLP-Recombinase/FRT directed insertion, partnered with a geneticin (G418) resistance cassette (HEK293T-R1872W). The latter enabled antibiotic selection post-integration, thereby establishing a stabilized R1872W cell line conducive to the swift screening of sgRNA sequences. A heterozygote CHO cell line (Invitrogen; Flp-In™-CHO Cell Line) containing the R1872W variant was created via the same transfection protocol (CHO-R1872W). Culture of CHO-R1872W and HEK293T-R1872W cells was performed according to previously published protocols (19). Cells were maintained in Dulbecco’s Modified Eagle medium (DMEM; Life Technologies) supplemented with 10% fetal bovine serum (Gibco), 0.1 mM nonessential amino acids (NEAA, Life Technologies), Glutamax (GM, Life Technologies).

Cloning and Viral Packaging

The sgRNA expression plasmid pU6-pegRNA-GG-acceptor (Addgene Plasmid #132777) was subjected to mRFP1 removal and replacement of a GFP cassette partnered with a self-cleaving P2A peptide. The GFP-P2A insertion enables the guide RNA to be transcribed independently of GFP. This allowed for rapid assessment of transfection efficiency mediated by the guide RNA plasmid. Subsequent individual sgRNAs were then cloned into the sgRNA expression plasmid (sgRNA-P2A-GFP). Constructs were transformed into Top10 chemically competent *Escherichia coli* (Thermo Fisher) grown on LB agar plates, and liquid cultures were grown in LB broth overnight at 37°C with 100 µg/ml ampicillin. Individual colonies were validated by Sanger sequencing (Genscript Inc). Verified plasmids were prepared by maxiprep (Qiagen).

Base Editor Construct Characterization

PCR was performed at the following conditions:

Category	Details
Forward Primer	CATGTACATCGCCATTATTTTG
Reverse Primer	CTTTGTCACGCTGTCGTAAGAG

Step	Temperature
1	94°C
2	94°C
3	60°C
4	68°C
5	–
6	68°C
7	12°C

Sanger Primer CTCGGTGGGTGCCTCCATTCTC

Mouse Husbandry and Genotyping

Scn8a$^{W/+}$ mice were generated as previously described (10,32) and maintained through crosses with C57BL/6J mice (Jax, #000664) to keep all experimental mice on a C57BL/6J genetic background (8,10). Cell type-specific expression of R1872W was achieved using males heterozygous for the R1872W allele and C57BL/6J females homozygous for either EIIa-Cre (Jax, #017320) or EMX1-Cre (Jax 005628) to generate mutant mice (*Scn8a*$^{W/+}$-EIIa and *Scn8a*$^{W/+}$-EMX1) (10).

Next Generation Amplicon Genomic DNA Sequencing

Category	Details
First Forward Primer (FP1)	CATGTACATCGCCATTATTTTG
First Reverse Primer (RP1)	CTTTGTCACGCTGTCGTAAGAG

Step	Temperature
1	94°C
2	94°C
3	60°C
4	68°C
5	-

6	68°C
7	12°C

Second Forward Primer (FP2) CAAGCCCAACACCATCGAG

Second Reverse Primer (RP2) CTTTGGAAGGATTGGACGCC

Step	Temperature
1	94°C
2	94°C
3	60°C
4	68°C
5	–
6	68°C
7	12°C

Adapter primers for Illumina sequencing were created and amplified by Azenta ('Genewiz") Inc. (Forward Primer: 5'-ACACTCTTTCCCTACACGACGCTCTTCCGATCT-3'; Reverse Primer: 5'GACTGGAGTTCAGACGTGTGCTCTTCCGATCT-3').

Supplemental Figures

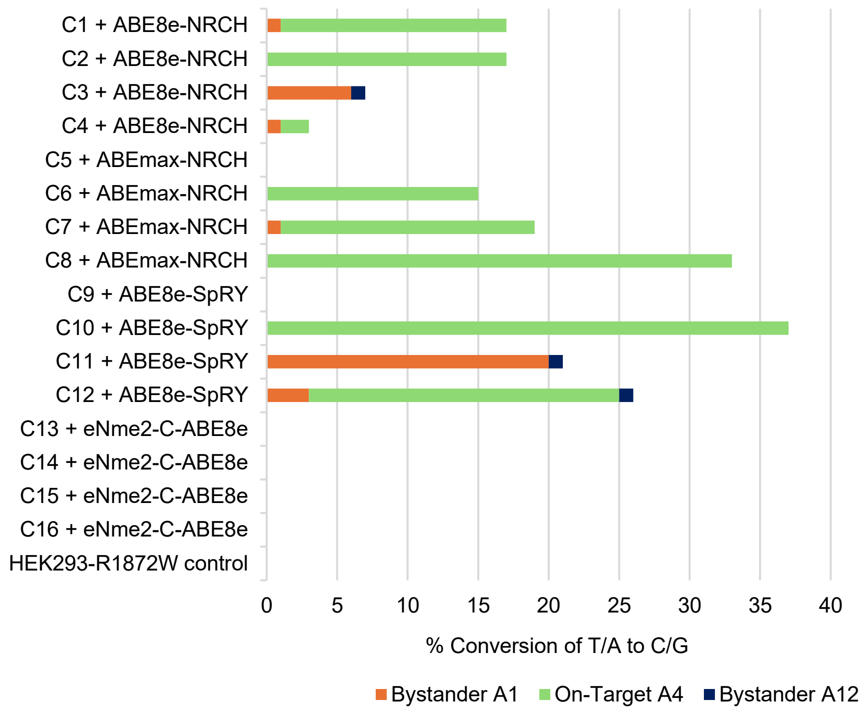

Supplemental Figure 1. ABE efficiencies of 16 constructs in R1872W expressing HEK293 cells. Results of Sanger sequencing for on-target (A4, green) and bystander (A1 and A12; orange and navy respectively) A-to-G editing efficiencies for base editor constructs within the targeted sequence. HEK293-R1872W control cells showed 2% signal at A12 when compared to *SCN8A*-ABE experimental groups. X-axis shows percent editing on the negative strand of the R1872W locus. Analysis of files were conducted with EditR1.0.10 with default parameters.

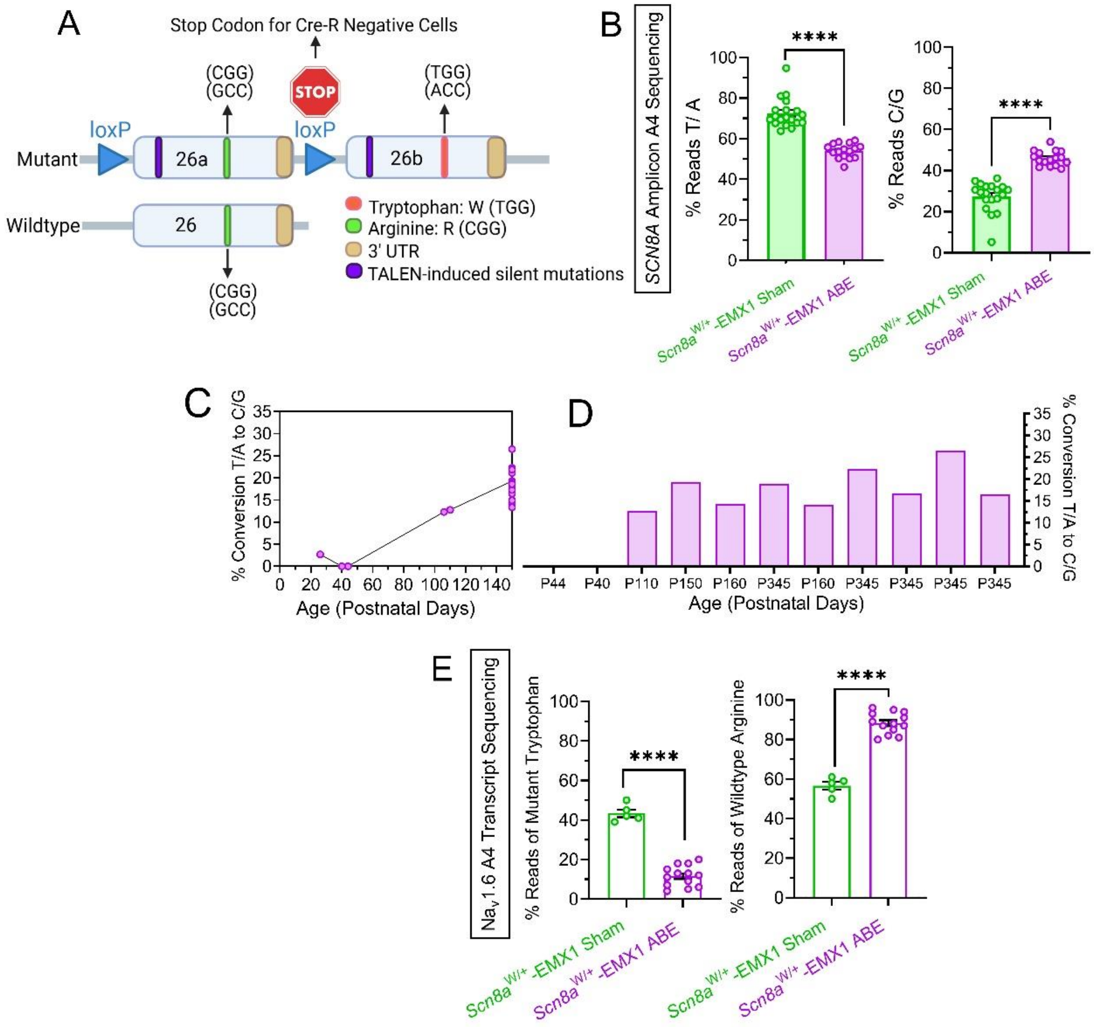

Supplemental Figure 2. ABE-mediated editing and survival outcomes in *Scn8a*$^{W/+}$-EMX1 mice

(**A**) The mutant chromosome carries two copies of the last exon, designated 26a and 26b, which differ in codon 1872 (10). Exons 26a and 26b carry 8 silent base substitutions (purple bar) and loxP sites flanking exon 26a (blue triangles). DNA sequencing was carried out by amplification with primers FP1 and FP2. A stop codon is located between exons 26a and 26b (red STOP symbol) results in EMX1-Cre negative (Cre-R Negative) cells only expressing 26a of the altered chromosome in on-target RNA transcript sequencing even as 26b remains unexcised. (**B**) Percentage of mutant T/A (left) and wildtype C/G (right) conversion in the genome of *Scn8a*$^{W/+}$-EMX1 *SCN8A*-ABE treated mice (n = 16, purple) and sham control (n = 19, green) mice (****, P< 0.0001; left, Mann-Whitney test; right, unpaired t-test). Each data point represents an individual mouse showing base pair identity. (**C**) Longitudinal analysis of T-to-C conversion at the R1872W allele plotted against survival time in *Scn8a*$^{W/+}$-EMX1 *SCN8A*-ABE treated mice (n = 21). Each point represents an individual mouse. (**D**) Percentage of T-to-C conversion on the mutant R1872W allele in *Scn8a* $^{W/+}$-EMX1 *SCN8A*-ABE treated mice (n = 11) from Figure 2.C at varying survival time points. Each bar represents an individual mouse. (**E**) Percentage of mutant tryptophan (left) to wildtype arginine (right) conversion at A4 R1872W on-target loci based on transcriptome-wide RNA-seq reads in *Scn8a*$^{W/+}$-EMX1 *SCN8A*-ABE treated mice (n = 13, purple) and *Scn8a*W/+-EMX1 sham control (n = 5, green) mice (****, P< 0.0001; unpaired t-test). Each data point represents an individual mouse showing R1872W Na_V1.6 codon transcript identity.

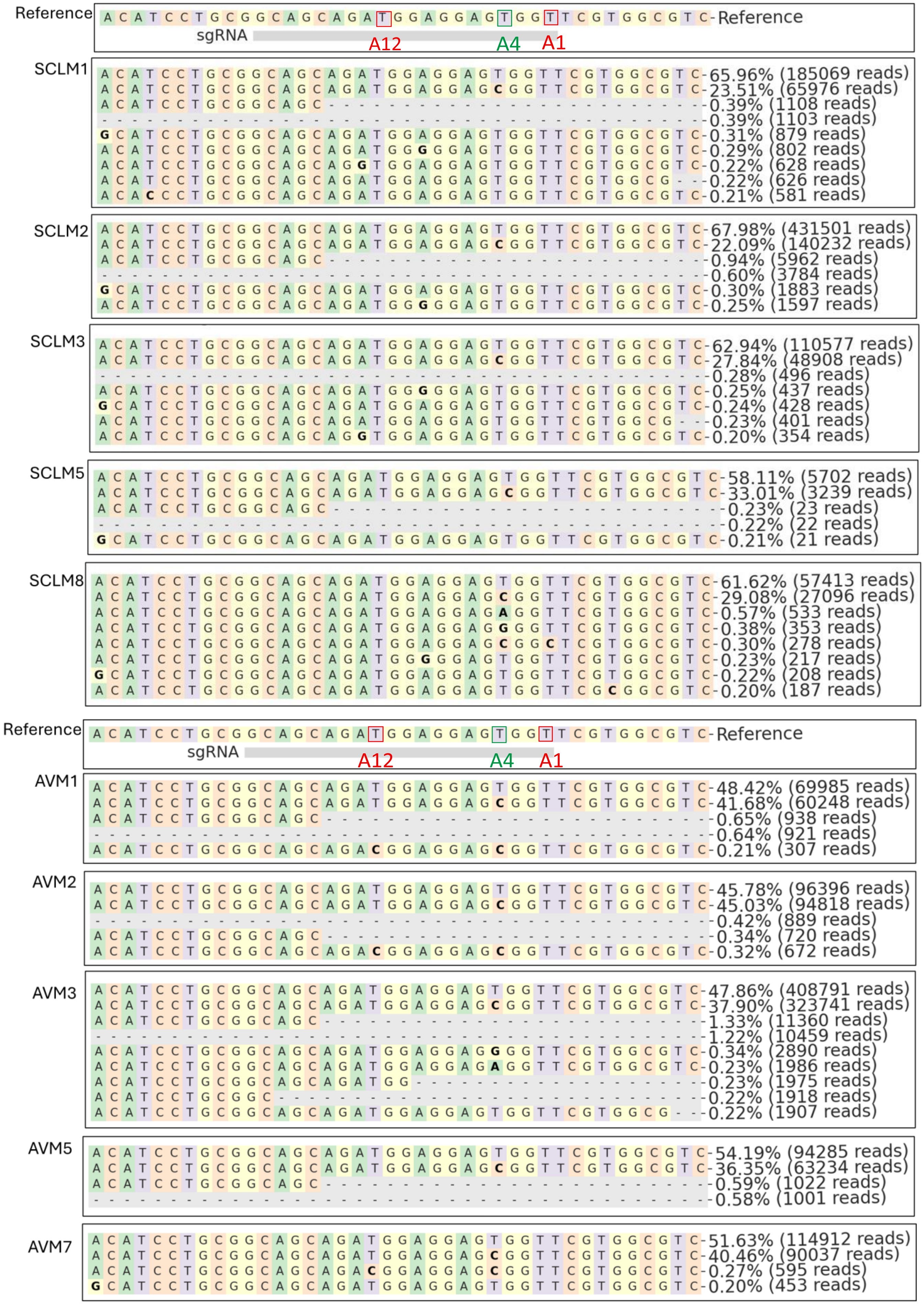

Reference
ACATCCTGCGGCAGCAGATGGAGGAGTGGTTCGTGGCGTC-Reference
sgRNA
A12
A4
A1
SCLM1
-65.96% (185069 reads)
-23.51% (65976 reads)
-0.39% (1108 reads)
-0.39% (1103 reads)
-0.31% (879 reads)
-0.29% (802 reads)
-0.22% (628 reads)
-0.22% (626 reads)
-0.21% (581 reads)
SCLM2
-67.98% (431501 reads)
-22.09% (140232 reads)
-0.94% (5962 reads)
-0.60% (3784 reads)
-0.30% (1883 reads)
-0.25% (1597 reads)
SCLM3
-62.94% (110577 reads)
-27.84% (48908 reads)
-0.28% (496 reads)
-0.25% (437 reads)
-0.24% (428 reads)
-0.23% (401 reads)
-0.20% (354 reads)
SCLM5
-58.11% (5702 reads)
-33.01% (3239 reads)
-0.23% (23 reads)
-0.22% (22 reads)
-0.21% (21 reads)
SCLM8
-61.62% (57413 reads)
-29.08% (27096 reads)
-0.57% (533 reads)
-0.38% (353 reads)
-0.30% (278 reads)
-0.23% (217 reads)
-0.22% (208 reads)
-0.20% (187 reads)
Reference
sgRNA
A12
A4
A1
AVM1
-48.42% (69985 reads)
-41.68% (60248 reads)
-0.65% (938 reads)
-0.64% (921 reads)
-0.21% (307 reads)
AVM2
-45.78% (96396 reads)
-45.03% (94818 reads)
-0.42% (889 reads)
-0.34% (720 reads)
-0.32% (672 reads)
AVM3
-47.86% (408791 reads)
-37.90% (323741 reads)
-1.33% (11360 reads)
-1.22% (10459 reads)
-0.34% (2890 reads)
-0.23% (1986 reads)
-0.23% (1975 reads)
-0.22% (1918 reads)
-0.22% (1907 reads)
AVM5
-54.19% (94285 reads)
-36.35% (63234 reads)
-0.59% (1022 reads)
-0.58% (1001 reads)
AVM7
-51.63% (114912 reads)
-40.46% (90037 reads)
-0.27% (595 reads)
-0.20% (453 reads)

Supplemental Figure 3: NGS amplicon bystander adenine confirmations in off-target analyzed mice. Standard CRISPResso2 (http://crispresso2.pinellolab.org/submission) sequencing read outcomes of 5 *Scn8a*$^{W+}$-EMX1 sham control mice (SCLM) compared to 5 *Scn8a*$^{W+}$-EMX1 *SCN8A*-ABE treated mice (AVM) from parallel survival studies. The gray horizontal bar indicates the sgRNA sequence reference sequence above both groups. All mice from each group underwent transcriptome-wide RNA-seq, and 2 mice from each group were included in WGS. Outputs from CRISPResso2 demonstrate no significant (≤0.5%) A1 and A12 bystander A-to-G activity in *SCN8A*-ABE mice compared to sham controls. Noise confirmation of deletions and substitutions were determined by direct comparison of *Scn8a*$^{W+}$-EMX1 *SCN8A*-ABE reads to *Scn8a*$^{W+}$-EMX1 sham control reads to discern significant editing events.

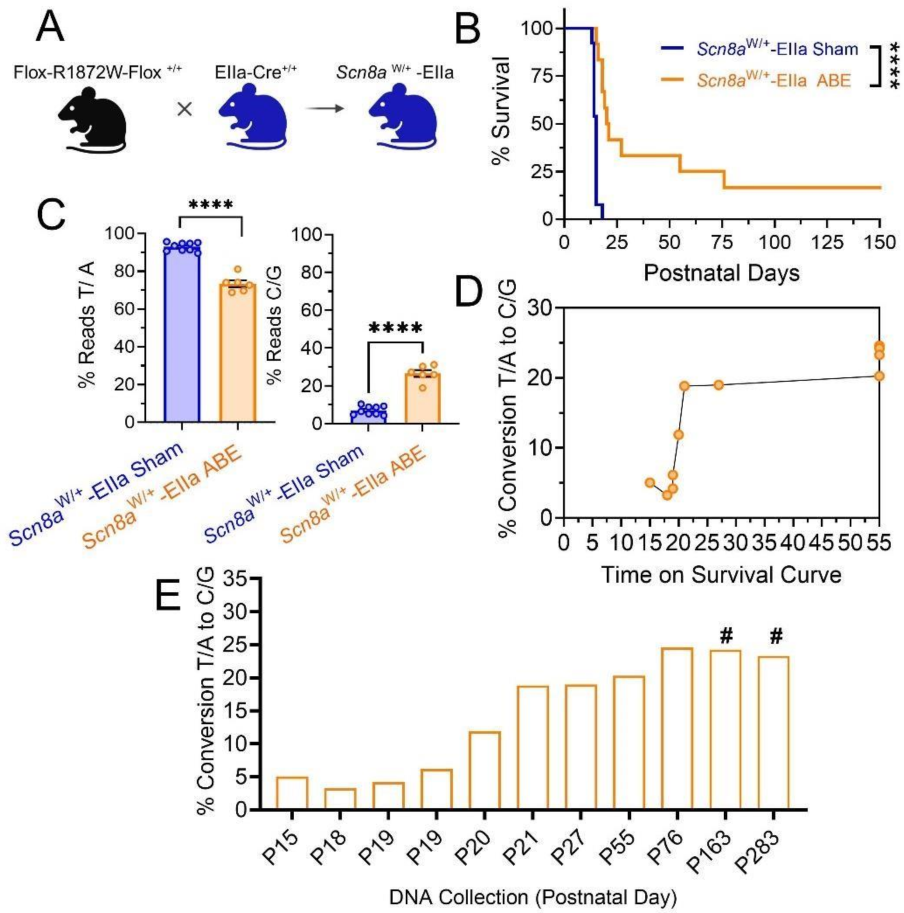

Supplemental Figure 4. ABE-mediated editing and survival outcomes in *Scn8a*$^{W/+}$-EIIa mice (**A**) Breeding strategy to generate *Scn8a*$^{W/+}$-EIIa mice. (**B**) Survival of *Scn8a*$^{W/+}$-EIIa mice is significantly increased with *SCN8A*-ABE treatment (orange, n = 12) compared with sham control *Scn8a*$^{W/+}$-EIIa mice (blue, n = 13, *P*<0.0001, Log-rank Mantel-Cox test). (**C**) Percentage of mutant T/A (left) and wildtype C/G (right) conversion in the genome of *Scn8a*$^{W/+}$-EIIa *SCN8A*-ABE treated mice (n = 6, orange) compared to *Scn8a*$^{W/+}$-EIIa sham control mice (n = 9, blue; ****, *P*< 0.0001; Welch's t-test). Each data point represents an individual mouse showing R1872W base pair identity. (**D**) Longitudinal analysis of T-to-C % conversion at the R1872W allele plotted against survival time in *Scn8a*$^{W/+}$-EIIa *SCN8A*-ABE treated mice (n = 11). Each point represents an individual mouse. (**E**) Percentages of each mouse of ABE-mediated T-to-C % conversion on the mutant *SCN8A* R1872W allele in *Scn8a* $^{W/+}$-EIIa *SCN8A*-ABE treated mice (n = 11) at different survival time points. # denotes mice that were euthanized for sequencing purposes. No symbol above the bar indicates mice that were found dead immediately before sample was collected. Data represents mean ± S.E.M.

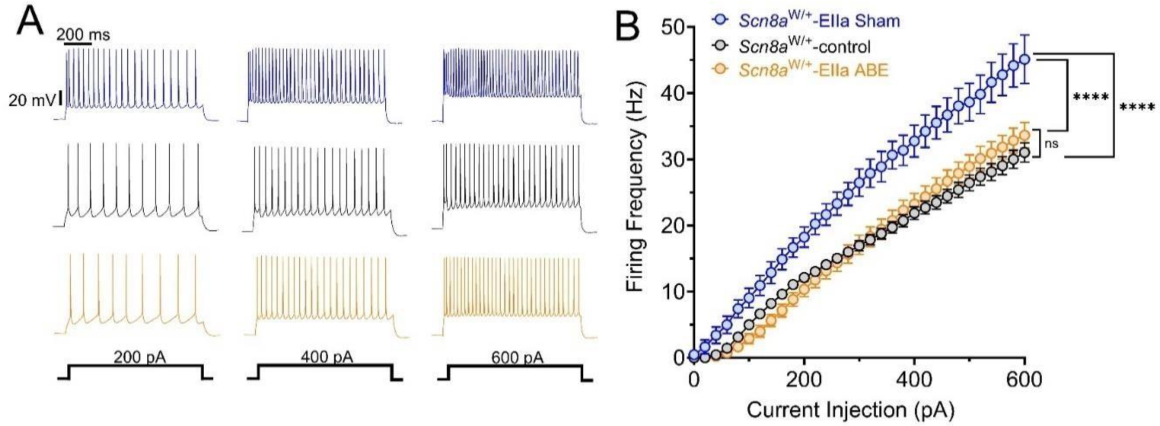

Supplemental Figure 5. *SCN8A*-ABE attenuates neuronal hyperexcitability in *Scn8a*$^{W/+}$-EIIa mice.
(**A**) Example traces of action potential firing from *Scn8a*$^{W/+}$-EIIa Sham (blue), *Scn8a*$^{W/+}$-control (black), and *Scn8a*$^{W/+}$-EIIa ABE (orange) neurons in somatosensory cortex layers IV/V at current injection steps of 200 pA, 400 pA, or 600 pA (P13-P17). (**B**) *Scn8a*$^{W/+}$-EIIa Sham (n = 17, 5 mice) neurons exhibit increased firing when compared to *Scn8a*$^{W/+}$-control (n = 29, 5 mice) that is attenuated in *Scn8a*$^{W/+}$-EIIa ABE neurons (n = 26 cells, 8 mice; ****, P<0.0001; Nested One-Way Anova with Dunns's multiple comparisons test). Data represents mean ± S.E.M.

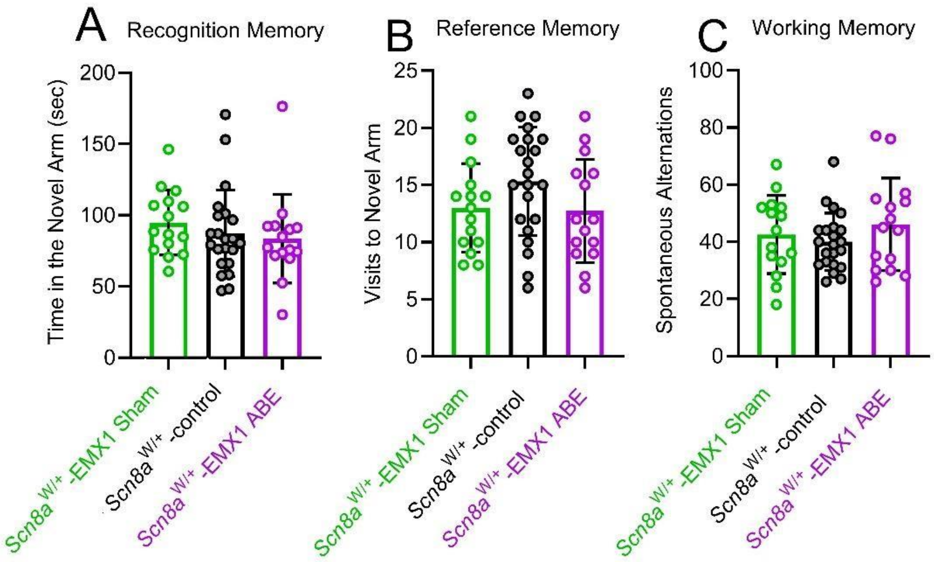

Supplemental Figure 6. *R1872W* mutation does not affect general cognition and memory.
(**A**) Recognition memory task, (**B**) reference memory task, or (**C**) working memory test were not different between *Scn8a*$^{W/+}$-EMX1 sham control (n = 15, green), *Scn8a*$^{W/+}$-control (n = 22), and *Scn8a*$^{W/+}$-EMX1 *SCN8A*-ABE treated mice (n = 15, purple). Data analyzed by one-way ANOVA with Dunns's multiple comparisons test. Each data point represents an individual mouse. Bars represent mean ± S.E.M

Guide ID	Guide RNA Sequence	PAM	Construct	Bystander A1	On-Target A4	Bystander A12	Additional Bystander As
**C1**	GAACCACTCCTCCATCTGCTGC	CGCA	ABE8e-NRCH.v1	1	16	0	0
**C2**	GACCACTCCTCCATCTGCTGCC	CGCA	ABE8e-NRCH.v2	0	17	0	0
**C3**	GCGAACCACTCCTCCATCTGC	TGCC	ABE8e-NRCH.v3	6	0	0	0
**C4**	GGAACCACTCCTCCATCTGC	TGCC	ABE8e-NRCH.v4	1	2	0	0
**C5**	GAACCACTCCTCCATCTGCTGC	CGCA	ABEmax-NRCH.v1	0	0	0	0
**C6**	GACCACTCCTCCATCTGCTGCC	CGCA	ABEmax-NRCH.v2	0	15	0	0
**C7**	GCGAACCACTCCTCCATCTGC	TGCC	ABEmax-NRCH.v3	1	18	0	0
**C8**	GGAACCACTCCTCCATCTGC	TGCC	ABEmax-NRCH.v4	0	33	0	0
**C9**	GAACCACTCCTCCATCTGCTG	CCG	ABE8e-SpRY.v1	0	0	0	0
**C10**	GAACCACTCCTCCATCTGCT	GCC	ABE8e-SpRY.v2	0	37	0	0
**C11**	GACCACTCCTCCATCTGCTGCC	GCA	ABE8e-SpRY.v4	20	0	0	0
**C12**	GCGAACCACTCCTCCATCTG	CTG	ABE8e-SpRY.v5	3	22	0	0
**C13**	GACGCCACGAACCACTCCTCCA	TCTGCT	eNme2-C-ABE8e.v1	0	0	0	0
**C14**	GCCACGAACCACTCCTCCATCTG	GCTGCC	eNme2-C-ABE8e.v2	0	0	0	0
**C15**	GCGCCACGAACCACTCCTCCATCT	CTGCCG	eNme2-C-ABE8e.v3	0	0	0	0
**C16**	GCACGAACCACTCCTCCATCTGCT	GCCGCA	eNme2-C-ABE8e.v4	0	0	0	0

Supplemental Table 1: R1872W targeting guide RNA sequences and ABEs for cell screen assays. A panel of the 16 chosen guide RNA (gRNA) sequences paired with a specific base editor construct targeting the R1872W mutation was designed for base editing. Each gRNA sequence is associated with a PAM motif. The adenine highlighted in green depicts the on target adenine, A4, that results in the generation of the R1872W mutation; adenines highlighted in red depict bystander positions relative to the on target A4 adenine.

**Mouse ID**	**Seizure Frequency**	**DNA Editing Efficiency (NGS, %)**	**RNA Editing Efficiency (RNA-seq, %)**
**1**	0	19	38
**2**	0	22	39
**3**	8	15	25
**4**	0	17	36
**5**	0	22	28
**6**	0	18	24
**7**	3	13	23
**8**	0	21	30
**9**	2	3	-
**10**	12	12	-
**11**	0	ND	ND

Supplemental Table 2: Correlation between transcript expression and seizure reduction or seizure inhibition. In *SCN8A*-ABE treated mice, a reduction of mutant transcripts ≥ 18% leads to seizure suppression. ND – Not Determine; mouse alive at P365.

Na$_v$ Subunit	Gene Name (Mouse)	RGEN Tools with up to 8 Mismatches	Number of Mismatches to Guide	Region	RNA Seq Editing	DNA Seq OFF
**Na$_v$1.1**	*Scn1a*	No Alignments	-	N/A	N/A	N/A
**Na$_v$1.2**	*Scn2a*	chr2:65756170	4	Intron noncoding	N/A	(No Activity)
**Na$_v$1.3**	*Scn3a*	chr2:65471458	4	Intron noncoding	N/A	(No Activity)
**Na$_v$1.4**	*Scn4a*	chr11:106319975	4	Exon coding	N/A, Not Expressed in Brain	N/A
**Na$_v$1.5**	*Scn5a*	No Alignments	-	N/A	N/A	N/A
**Na$_v$1.6**	*Scn8a*	chr15:101040338	1 Mismatch: 5'G for U6 transcription	Exon coding	31%	21%
**Na$_v$1.7**	*Scn9a*	chr2:66574826	5	Intron noncoding	N/A	(No Activity)
**Na$_v$1.8**	*Scn10a*	chr9:119692037	4	Intron noncoding	N/A	(No Activity)
**Na$_v$1.9**	*Scn11a*	chr9:1198 01862	4	Intron noncoding	N/A	(No Activity)

Supplemental Table 3: Off-target results from voltage gated sodium channel family genes and transcripts. Summary of A-to-G off-target assessments for all voltage-gated sodium (Nav) channel family genes and subunits in mouse, using guide RNA designed for *SCN8A* (Na$_v$1.6). RGEN tool analyses were used to identify potential off-target loci with up to 9 mismatches. Transcriptome wide RNA-seq and WGS data indicate no detectable off-target editing activity at these regions. (-) Number of mismatches to guide corresponds to no homology alignments detected.

Supplemental Table 4: Membrane, AP, and channel properties of *Scn8a*$^{W/+}$-EMX1 neurons

	V_m (mV)	AP Threshold (mV)	Rheobase (pA)	Upstroke Velocity (mV/ms)	Downstroke Velocity (mV/ms)	Amplitude (mV)	APD_{50} (ms)	Input Resistance (MΩ)	$V_{1/2}$ (mV)
***Scn8a*$^{W/+}$-control (*n*=21, 4; *n*=17, 6)**	-67.0 ± 1.0	-46.7 ± 0.8	126.7 ± 14.1	283.4 ± 13.9	-64.7 ± 6.9	91.5 ± 2.5	1.4 ± 0.1	101.8 ± 8.8	-58.0 ± 2.4
***Scn8a*$^{W/+}$-EMX1 ABE (*n*=33, 7; *n*=17, 8)**	-67.4 ± 0.9	-45.0 ± 0.6	173.5 ± 12.3 *	389.3 ± 20.8 ***	-122.0 ± 6.9 ****	83.2 ± 1.8 *	0.84 ± 0.0 ****	76.9 ± 4.6*	-61.0 ± 1.7
***Scn8a*$^{W/+}$-EMX1 Sham (*n*=25, 4; *n*=24, 7)**	-66.1 ± 0.9	-46.0 ± 0.7	152.0 ± 10.7	382.3 ± 23.4	-98.0 ± 5.5 **, #	88.0 ± 1.7	1.0 ± 0.1 **	81.7 ± 5.5	-61.6 ± 1.7

Recordings were carried out in pyramidal neurons from Layer 4/5 somatosensory cortex of *Scn8a*$^{W/+}$-control, *Scn8a*$^{W/+}$-EMX1 *SCN8A*-ABE treated, and *Scn8a*$^{W/+}$-EMX1 sham control mice at P17-25. Recordings were performed in multiple cells from each animal (*n*=cells, animals) and in multiple experiments as shown here: genotype (membrane/AP properties *n*; channel properties *n*).

*, $P<0.05$; **, $P<0.01$; ***, $P<0.001$; ****, $P<0.0001$ indicate significance vs. *Scn8a*$^{W/+}$-control mice.

#, $P<0.05$; ##, $P<0.01$; ####, $P<0.0001$ indicate significance vs. *Scn8a*$^{W/+}$-EMX1 *SCN8A*-ABE treated mice. Data is presented as mean ± SEM.

Supplemental Table 5: Membrane and AP properties of *Scn8a*$^{W/+}$-EIIa neurons

	V_m (mV)	AP threshold (mV)	Rheobase (pA)	Upstroke Velocity (mV/ms)	Downstroke Velocity (mV/ms)	Amplitude (mV)	APD_{50} (ms)	Input Resistance (MΩ)
***Scn8a*$^{W/+}$control (n=29,5)**	-64.7 ± 0.6	-43.9 ± 0.6	81.4 ± 6.9	269.5 ± 14.8	-62.0 ± 3.2	84.4 ± 2.3	1.4 ± 0.1	120.5 ± 5.8
***Scn8a*$^{W/+}$-EIIa ABE (*n*=26, 8)**	-67.4 ± 1.1	-46.7 ± 0.9 #	111.5 ± 9.4 *	377.2 ± 20.3 ***	-69.8 ± 4.4	86.9 ± 1.7	1.2 ± 0.1	110.4 ± 7.9
***Scn8a*$^{W/+}$-EIIa Sham (*n*=17, 5)**	-64.2 ± 0.9	-47.6 ± 1.5 *	72.9 ± 11.2 #	285.9 ± 17.0 #	-58.2 ± 5.0	89.1 ± 2.1	1.6 ± 0.1 ##	136.7 ± 11.0

Recordings were carried out in pyramidal neurons from Layer 4/5 somatosensory cortex of *Scn8a*$^{W/+}$-control, *Scn8a*$^{W/+}$-EIIa *SCN8A*-ABE treated, and *Scn8a*$^{W/+}$-EIIa sham control mice at P13-17. Recordings were performed in multiple cells from each animal (n=cells, animals). Data is presented as mean ± SEM.

*, *P*<0.05; ***, *P*<0.001 indicate significance when compared to *Scn8a*$^{W/+}$-control mice.

#, *P*<0.05; ##, *P*<0.01; ###, *P*<0.001 indicate significance when compared to *Scn8a*$^{W/+}$-EIIa *SCN8A*-ABE treated mice.
